# Plasma phospho-tau217 for Alzheimer’s disease diagnosis in primary and secondary care using a fully automated platform

**DOI:** 10.1038/s41591-025-03622-w

**Published:** 2025-04-09

**Authors:** Sebastian Palmqvist, Noëlle Warmenhoven, Federica Anastasi, Andrea Pilotto, Shorena Janelidze, Pontus Tideman, Erik Stomrud, Niklas Mattsson-Carlgren, Ruben Smith, Rik Ossenkoppele, Kübra Tan, Anna Dittrich, Ingmar Skoog, Henrik Zetterberg, Virginia Quaresima, Chiara Tolassi, Kina Höglund, Duilio Brugnoni, Albert Puig-Pijoan, Aida Fernández-Lebrero, José Contador, Alessandro Padovani, Mark Monane, Philip B. Verghese, Joel B. Braunstein, Silke Kern, Kaj Blennow, Nicholas J. Ashton, Marc Suárez-Calvet, Oskar Hansson

**Affiliations:** 1 Clinical Memory Research Unit, Department of Clinical Sciences Malmö, Faculty of Medicine, Lund University, Lund, Sweden; 2 Memory Clinic, Skåne University Hospital, Malmö, Sweden; 3 Barcelonaβeta Brain Research Center (BBRC), Pasqual Maragall Foundation, Barcelona, Spain; 4 Hospital del Mar Research Institute, Barcelona, Spain; 5 Centre for Genomic Regulation (CRG), Barcelona Institute of Science and Technology (BIST), Barcelona, Spain; 6 Neurology Unit, Department of Clinical and Experimental Sciences, University of Brescia, Brescia, Italy; 7 Department of Continuity of Care and Frailty, Neurology Unit, ASST Spedali Civili Hospital, Brescia, Italy; 8 Neurobiorepository and Laboratory of advanced biological markers, University of Brescia and ASST Spedali Civili Hospital, Brescia, Italy; 9 Wallenberg Center for Molecular Medicine, Lund University, Lund, Sweden; 10 Neurology, Vrije Universiteit Amsterdam, Alzheimer Center Amsterdam, Amsterdam, The Netherlands; 11 Amsterdam Neuroscience—Neurodegeneration, Vrijie Universiteit Amersterdam, Amsterdam, The Netherlands; 12 Institute of Neuroscience and Physiology, Department of Psychiatry and Neurochemistry, Sahlgrenska Academy at University of Gothenburg, Mölndal, Sweden; 13 Department of Neuropsychiatry, Region Västra Götaland, Sahlgrenska University Hospital, Mölndal, Sweden; 14 Clinical Neurochemistry Laboratory, Sahlgrenska University Hospital, Mölndal, Sweden; 15 Department of Neurodegenerative Disease, UCL Queen Square Institute of Neurology, London, UK; 16 UK Dementia Research Institute at UCL, London, UK; 17 Hong Kong Center for Neurodegenerative Diseases, Hong Kong, China; 18 Wisconsin Alzheimer’s Disease Research Center, University of Wisconsin School of Medicine and Public Health, University of Wisconsin–Madison, Madison, WI USA; 19 Residency Program in Clinical Pathology and Clinical Biochemistry, Department of Molecular and Translational Medicine, University of Brescia, Brescia, Italy; 20 Central Laboratory for Clinical Chemistry, ASST Spedali Civili Hospital, Brescia, Italy; 21 Brain Health Center, University of Brescia, Brescia, Italy; 22 Department of Clinical Genetics and Genomics, Center for Medical Genomics, Sahlgrenska University Hospital, Gothenburg, Sweden; 23 Servei de Neurologia, Hospital del Mar, Barcelona, Spain; 24 Department of Medicine, Universitat Autònoma de Barcelona, Barcelona, Spain; 25 Department of Medicine and Life Sciences, Universitat Pompeu Fabra, Barcelona, Spain; 26 C2N Diagnostics LLC, St. Louis, MO USA; 27 Paris Brain Institute, ICM, Pitié-Salpêtrière Hospital, Sorbonne University, Paris, France; 28 Neuroscience Maurice Wohl Clinical Neuroscience Institute, King’s College London, Institute of Psychiatry, London, UK; 29 Banner Alzheimer’s Institute, Phoenix, AZ USA; 30 Banner Sun Health Research Institute, Sun City, AZ USA

**Keywords:** Diagnostic markers, Alzheimer's disease

## Abstract

Global implementation of blood tests for Alzheimer’s disease (AD) would be facilitated by easily scalable, cost-effective and accurate tests. In the present study, we evaluated plasma phospho-tau217 (p-tau217) using predefined biomarker cutoffs. The study included 1,767 participants with cognitive symptoms from 4 independent secondary care cohorts in Malmö (Sweden, *n* = 337), Gothenburg (Sweden, *n* = 165), Barcelona (Spain, *n* = 487) and Brescia (Italy, *n* = 230), and a primary care cohort in Sweden (*n* = 548). Plasma p-tau217 was primarily measured using the fully automated, commercially available, Lumipulse immunoassay. The primary outcome was AD pathology defined as abnormal cerebrospinal fluid Aβ42:p-tau181. Plasma p-tau217 detected AD pathology with areas under the receiver operating characteristic curves of 0.93–0.96. In secondary care, the accuracies were 89–91%, the positive predictive values 89–95% and the negative predictive values 77–90%. In primary care, the accuracy was 85%, the positive predictive values 82% and the negative predictive values 88%. Accuracy was lower in participants aged ≥80 years (83%), but was unaffected by chronic kidney disease, diabetes, sex, *APOE* genotype or cognitive stage. Using a two-cutoff approach, accuracies increased to 92–94% in secondary and primary care, excluding 12–17% with intermediate results. Using the plasma p-tau217:Aβ42 ratio did not improve accuracy but reduced intermediate test results (≤10%). Compared with a high-performing mass-spectrometry-based assay for percentage p-tau217, accuracies were comparable in secondary care. However, percentage p-tau217 had higher accuracy in primary care and was unaffected by age. In conclusion, this fully automated p-tau217 test demonstrates high accuracy for identifying AD pathology. A two-cutoff approach might be necessary to optimize performance across diverse settings and subpopulations.

## Abstract (web summary)

Among 1,767 patients in 5 centers, a fully automated blood test showed high accuracy for Alzheimer’s pathology in secondary and primary care using a predefined cutoff.

## Main

In recent years, notable progress has been made in identifying blood-based biomarkers (BBMs) for detecting Alzheimer’s disease (AD) pathology in symptomatic patients^1^. Among these BBMs, phosphorylated tau217 (p-tau217), measured alone or as a ratio relative to the concentration of nonphosphorylated tau217 (np-tau217), or Aβ42, has demonstrated superior performance across various methods, including immunoassays and mass spectrometry (MS)-based assays^2–7^. Despite these promising results, there are still a few challenges for the real-world implementation of BBMs into routine clinical practice. Some studies suggest that MS-based assays have high accuracy, comparable to US Food and Drug Administration (FDA)-approved cerebrospinal fluid (CSF) assays^4,8^, but their use is currently restricted to highly specialized laboratories. Conversely, fully automated immunoassay platforms offer a practical and scalable solution, leveraging the capacity of most clinical routine laboratories, which already rely on such platforms for diagnosing numerous disorders. However, there is a need to validate fully automated immunoassays for AD to make them more generally available in different clinical settings.

Diagnosis of AD is challenging, with up to 35% of patients potentially being misclassified in specialized clinics and 39% in primary care, when AD biomarkers are not used^9,10^. Most AD diagnoses are made by nonspecialist clinicians, such as primary care physicians, who are often the first to assess patients with cognitive symptoms but frequently have no access to currently available AD biomarkers, namely, CSF analysis or amyloid positron emission tomography (PET). The introduction of fully automated immunoassays may improve accessibility to these new biomarkers.

As anti-amyloid treatments for early symptomatic AD have become available in some countries, accurately identifying the disease’s etiology has become increasingly important because these treatments require biomarker confirmation of amyloid pathology. Furthermore, AD is often diagnosed in later stages, limiting opportunities for early intervention when the treatments are most effective. BBMs, with their broader accessibility, scalability and patient acceptability, hold the potential to provide more individuals with accurate and timely diagnoses, ensuring equitable access to appropriate care and new medications.

We have learned from CSF biomarkers that fully automated assays are essential for ensuring reproducibility of the results, particularly to facilitate the global adoption of these biomarkers^11^. A widely used CSF assay in clinical practice is the FDA-approved Lumipulse assay^12^. Recently, a plasma p-tau217 immunoassay was launched on the fully automated Lumipulse platform. Our aim was to validate this plasma p-tau217 assay for detecting AD pathology in clinical settings. Specifically, we established biomarker cutoffs in one cohort, applied them to patient samples that had been collected from independent primary and secondary care cohorts from different countries and examined the impact of comorbidities and demographic factors on the plasma p-tau217 performance. As secondary analyses, we compared the performance of plasma p-tau217 with that of the p-tau217:Aβ42 (Lumipulse) ratio currently under FDA evaluation^13^ and a high-performing MS-based method for plasma %p-tau217 (p-tau217:np-tau217 × 100) currently being used in clinical practice in the United States of America^14^.

## Results

### Participants and biomarker characteristics

**Fig. 1:**
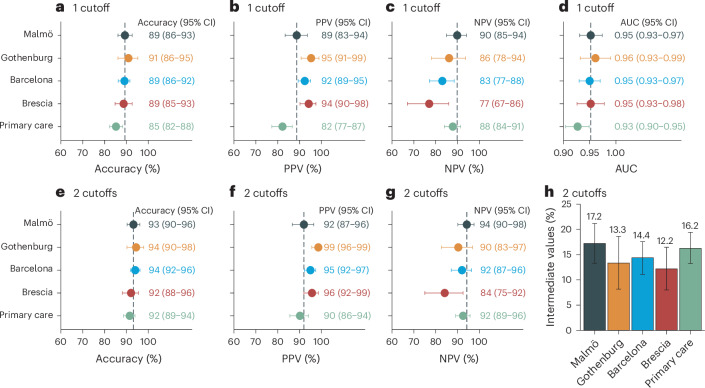
Performance of plasma p-tau217 (Lumipulse) for discriminating AD pathology-positive versus AD pathology-negative participants in five independent cohorts. **a**–**c**,**e**–**g**, The single cutoff was set at >0.27 pg ml^−1^ (accuracy (**a**), PPV (**b**) and NPV (**c**)) and the two cutoffs at <0.22 and >0.34 pg ml^−1^ (accuracy (**e**), PPV (**f**) and NPV (**g**)). Comparisons between primary and pooled secondary care are shown in Supplementary Table 1. **d**, Note that the AUC values are independent of cutoffs. **h**, Participants who fell between the two cutoffs were classified as intermediate. Vertical dashed lines mark the performance in the Malmö cohort where the cutoffs were established. Data are presented as the observed percentage and the error bars as the 95% CI derived from the bootstrap distribution. AD pathology was defined as CSF Aβ42:p-tau181 < 11.94 or positive visual read on amyloid PET if lumbar puncture was not performed (*n*_missing_ = 87 in primary care). The AD pathology prevalence was *n* = 153+ or 84− in Malmö, 93+ or 72− in Gothenburg, 321+ or 166− in Barcelona, 164+ or 66− in Brescia and 244+ or 305− in primary care (Sweden). Accuracy indicates percentage of correctly classified participants.

**Extended Data Fig. 1:**
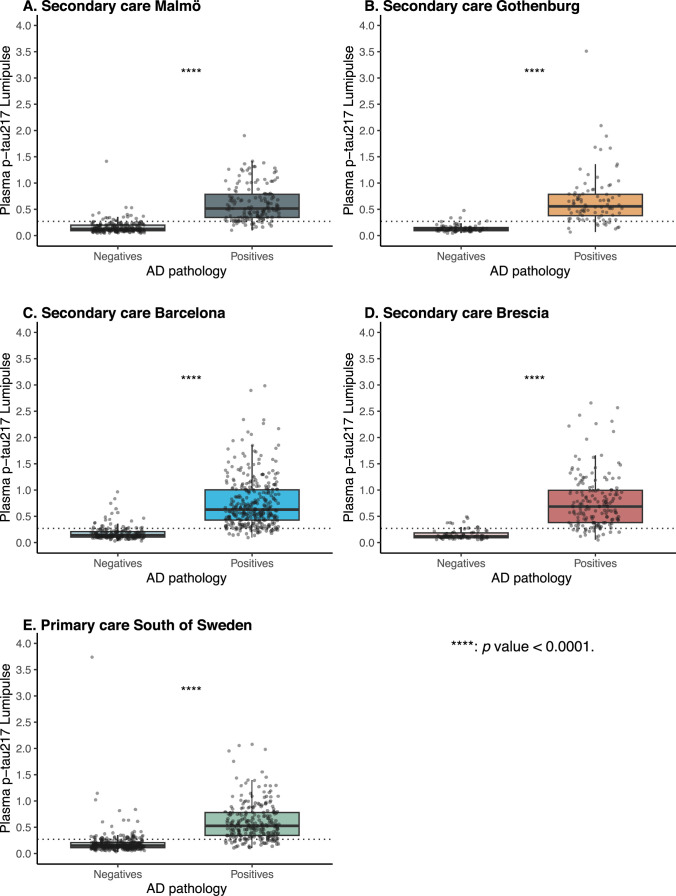
Boxplots of plasma p-tau217 (Lumipulse) concentrations in the different cohorts with AD pathology as grouping variable. All p-tau217 levels differed significantly (*P* < 0.0001) between AD pathology-positive and AD pathology-negative participants in all cohorts, namely, Malmö (*n* = 337) (**a**), Gothenburg (*n* = 165) (**b**), Barcelona (*n* = 487) (**c**), Brescia (*n* = 230) (**d**), and in primary care (Sweden) (*n* = 548) (**e**). Horizontal dashed lines represent the single cutoff at 90% specificity established in (**a**) Secondary care Malmö. The dots represent individual participants. The central band of the boxplot represents the group median, the box limits correspond to the first and third quartiles, and the whiskers represent the minimum/maximum value or the 1.5 interquartile range (IQR) from the box, whichever is smaller. AD status was defined as CSF Aβ42/p-tau181 < 11.94 or positive amyloid PET visual read for those who did not undergo lumbar puncture. Group mean levels were compared with Student’s t-test. Abbreviations: AD, Alzheimer’s disease; IQR, interquartile range.

Overall, 1,767 patients with cognitive symptoms were enrolled (*n* = 1,219 in secondary care; *n* = 548 in primary care). The mean age was 73 (s.d. 9) years, 53% were women and 55% had AD pathology (Table 1). Plasma p-tau217 concentrations were significantly higher in AD pathology-positive versus AD pathology-negative participants (Extended Data Fig. 1) and areas under the receiver operating characteristic (ROC) curves (AUCs) ranged from 0.93 to 0.96 across cohorts (Fig. 1d).

**Table 1:** Characteristics of the cohorts

Variable	Secondary care (Malmö), *n* = 337	Secondary care (Gothenburg), *n* = 165	Secondary care (Barcelona), *n* = 487	Secondary care (Brescia), *n* = 230	Primary care (Sweden), *n* = 548
**Age, years**	72 (9.4)	66 (8.1)	73 (6.0)	71 (8.7)	76 (6.9)
**Sex, no. women,** ***n*** **(%)**	152 (45.1)	84 (50.9)	282 (57.9)	132 (57.4)	280 (51.1)
**Education, years**	12 (3.7)	13 (3.3)	8 (4.2)	9 (3.8)	11 (3.2)
**MMSE**	24 (5.0)	24 (4.7)	21 (5.4)	23 (4.7)	26 (3.4)
**Cognitive stage,** ***n*** **(%)**					
**SCD**	65 (19.3)	4 (2.4)	30 (6.2)	0 (0)	151 (27.5)
**MCI**	163 (48.4)	126 (76.4)	167 (34.3)	157 (68.3)	245 (44.7)
**Dementia**	109 (32.3)	35 (21.2)	289 (59.3)	73 (31.7)	152 (27.7)
***APOE*** **ε4 carriers,** ***n*** **(%)**	154 (45.7)	99 (60.0)	167 (34.3)	104 (45.2)	236 (43.1)
**Plasma p-tau217 (pg** **ml**^−^^**1**^**)**^**a**^	0.36 (0.33)	0.44 (0.46)	0.56 (0.48)	0.60 (0.52)	0.38 (0.37)
**CSF Aβ42:40 ratio**	0.073 (0.027)	0.065 (0.025)	0.060 (0.025)	0.068 (0.08)^b^	0.069 (0.026)^c^
**CSF p-tau181 (pg** **ml**^−^^**1**^**)**	60.4 (40.2)	64.0 (42.0)	91.3 (58.3)	91.8 (62.2)	63.9 (40.4)^c^
**CSF Aβ42:p-tau181 ratio**	20.7 (15.1)	17.6 (14.2)	12.8 (11.6)	12.0 (19.1)	18.6 (13.0)^c^
**AD positive,** ***n*** **(%)**	153 (45.4)	93 (56.4)	321 (65.9)	164 (71.3)	244 (44.5)^d^

Data are mean (s.d.) if not otherwise specified.

^a^Measured using the Lumipulse assay.

^b^Missing for *n* = 71 in Brescia–secondary care.

^c^Missing for *n* = 87 for those who did not undergo lumbar puncture in primary care.

^d^CSF Aβ42:p-tau181 < 11.94 or positive amyloid PET visual read for those who did not undergo lumbar puncture (*n* = 87 in primary care).

MMSE, Mini-Mental State Examination.

### Accuracy of plasma p-tau217 (Lumipulse)

The rationale and methods for the cutoffs are provided in Methods. A single plasma cutoff was established in the Malmö cohort at 90% specificity (>0.27 pg ml^−1^), yielding an accuracy of 89% (95% confidence interval (CI), 86–93%) in the same cohort. Applying the cutoff to out-of-sample secondary care participants, the accuracy was 91% (95% CI = 86–95%) in Gothenburg, 89% (95% CI = 86–92%) in Barcelona and 89% (95% CI = 85–93%) in Brescia. In primary care (Sweden), the accuracy was 85% (95% CI = 82–88%) (Fig. 1a–c), which was significantly lower than in secondary care (Supplementary Table 1).

Next, we used a two-cutoff approach. The two cutoffs were established at 95% sensitivity and 95% specificity in the Malmö cohort (upper cutoff >0.34 pg ml^−1^, lower cutoff <0.22 pg ml^−1^). They yielded an accuracy of 93% (95% CI = 90–96%) in the same cohort with 17% in the intermediate zone (that is, between the upper and lower cutoffs and not included when calculating the accuracy). Applying these two cutoffs to out-of-sample cohorts resulted in an accuracy of 94% (95% CI = 90–98%) in Gothenburg, 94% (95% CI = 92–96%) in Barcelona and 92% (95% CI = 88–96%) in Brescia, with 12–14% in the intermediate zone (Fig. 1e–h). In primary care (Sweden), the accuracy was 92% (95% CI = 89–94%) with 16% in the intermediate zone. Using these two cutoffs, all positive predictive values (PPVs) and negative predictive values (NPVs) were ≥90%, except for the NPV in Brescia (84%, 95% CI = 75–92%), where it was reduced as a result of a high prevalence of AD pathology (71%; Table 1).

### Effects of demographic factors and comorbidities

**Extended Data Fig. 2:**
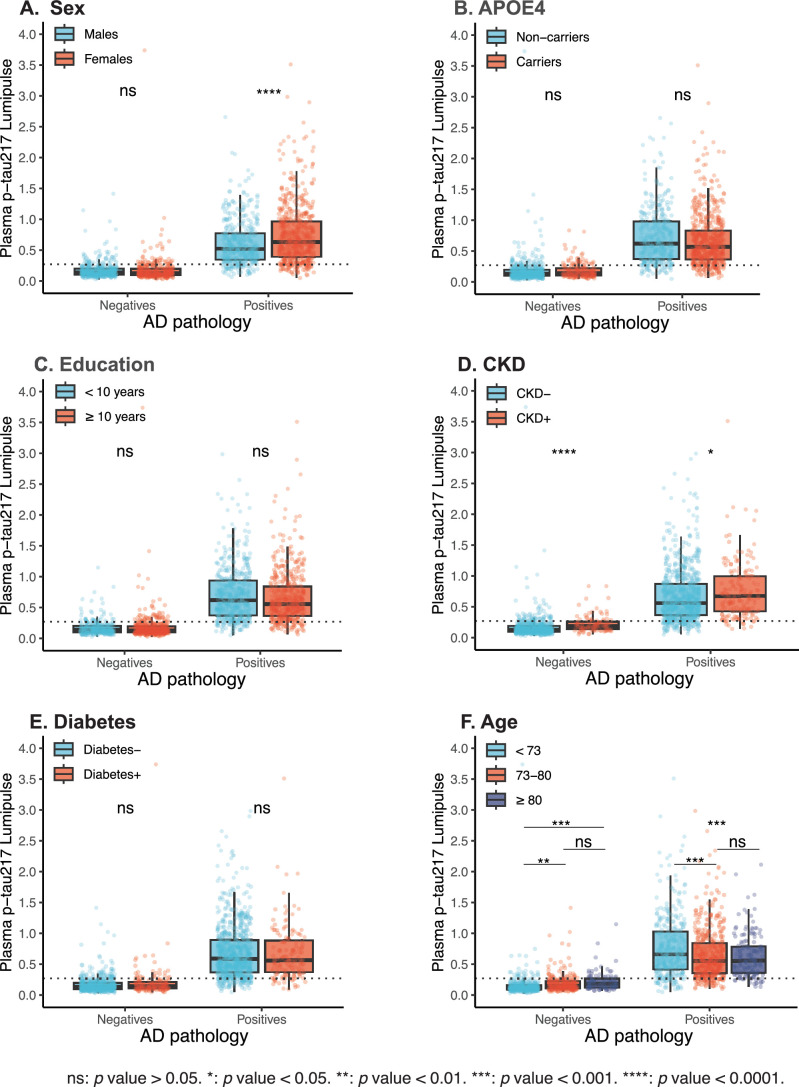
Plasma p-tau217 (Lumipulse) and the effect of demographic characteristics and comorbidities. Boxplots of plasma p-tau217 (Lumipulse) concentrations stratified by AD status and the following variables: (**a**) sex, (**b**) *APOE* ε4 carriership, (**c**) education, (**d**), chronic kidney disease, (**e**) presence of diabetes, and (**f**) age. The analyses were performed pooling the data of the five cohorts (*n* = 1767). Dashed lines represent the single cutoff at 90% specificity established in the Malmö secondary care cohort. The dots represent individual participants. The central band of the boxplot represents the group median, the box limits correspond to the first and third quartiles, and the whiskers represent the minimum/maximum value or the 1.5 interquartile range (IQR) from the box, whichever is smaller. AD status was defined as CSF Aβ42/p-tau181 < 11.94 or positive amyloid PET visual read for those who did not undergo lumbar puncture. Group mean levels were compared with Student’s t-test. Abbreviations: AD, Alzheimer’s disease; CKD, chronic kidney disease; IQR, interquartile range.

Among participants with AD pathology, plasma p-tau217 (Lumipulse) levels were significantly higher in women compared with men and higher in younger (aged <73 years) participants compared with older participants (aged 73–80 and ≥80 years) (Extended Data Fig. 2). Conversely, p-tau217 levels were lower in younger participants compared with older participants without AD pathology. In addition, p-tau217 concentrations were higher in participants with chronic kidney disease (CKD), irrespective of AD pathology status. There were no differences in p-tau217 concentrations when stratifying on *APOE* ε4 status (positivity defined as carrying at least one ε4 allele), educational level or presence of diabetes mellitus (Extended Data Fig. 2).

**Fig. 2:**
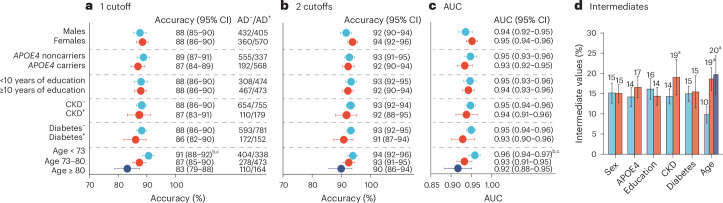
Effects of demographic factors and comorbidities on plasma p-tau217 (Lumipulse) performance. **a**–**c**, Accuracy using a single cutoff (**a**), two cutoffs (**b**) and AUC values (**c**). **d**, Participants with results between the two cutoffs classified as intermediate. The number of participants in each group, stratified by AD pathology, is indicated in **a**. Data are presented as the observed percentage and the error bars as the 95% CI derived from the bootstrap distribution. The analysis combined data from the five different cohorts (*n* = 1,767). The same analyses restricted to the primary care cohort can be found in Extended Data Fig. 3. AD pathology was defined as CSF Aβ42:p-tau181 < 11.94 or positive visual read on amyloid PET if lumbar puncture was not performed (*n* = 87). To assess whether the observed difference in the statistics is significantly different from zero, we performed a bootstrap hypothesis test. The *P* value (two sided) was calculated as the proportion of bootstrap resamples (*n* = 2,000) where the absolute null-distributed statistic was greater than or equal to the observed difference. Differences between AUCs were assessed using DeLong statistics. Results were not corrected for multiple comparisons. Significant *P* values in the order as presented in the plot: 0.046, 0.003 (**a**); 0.035, 0.026 (**c**); 0.034, <0.001, <0.001 (**d**). ^a^Significantly higher than group 1, *P* < 0.05. ^b^Significantly higher than group 2, *P* < 0.05. ^c^Significantly higher than group 3, *P* < 0.05.

**Extended Data Fig. 3:**
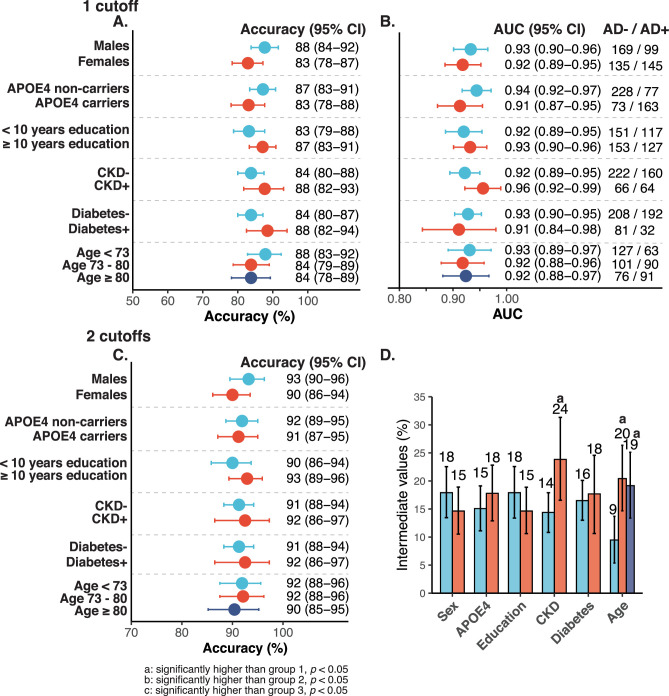
Effects of demographic characteristics and comorbidities on plasma p-tau217 performance in primary care. This figure presents the accuracy and predictive values of plasma p-tau217 (Lumipulse) across various subgroups to assess the impact of demographic characteristics and comorbidities in primary care only (*n* = 548). Results are shown using a single cutoff (**a**) and using two cutoffs (**c**). Note that the AUC is independent of cutoffs (**b**). Participants who fell between the two cutoffs were classified in the intermediate group (**d**). Data are presented as the observed percentage, and the error bars as the 95% CI derived from the bootstrap distribution. AD pathology was defined as CSF Aβ42/p-tau181 < 11.94 or positive visual read on amyloid PET if lumbar puncture was not performed. The AD pathology prevalence is depicted for each cohort per subgroup in the figure (**b**). To assess whether the observed difference in the statistics is significantly different from zero, we performed a bootstrap hypothesis test. The *P* value (two-sided) was calculated as the proportion of bootstrap resamples (n = 2000) where the absolute null-distributed statistic was greater than or equal to the observed difference. Differences between AUCs were assessed with DeLong statistics. Results were not corrected for multiple comparisons. Significant *P* values in the order as presented in the plot: 0.022; 0.002; 0.007. Abbreviations: Accuracy; percent correctly classified participants; CI, confidence interval; CKD, chronic kidney disease; NPV, negative predictive value; PPV, positive predictive value.

Despite the influence of some of these factors on p-tau217 concentrations, they did not significantly affect the accuracy of identifying AD pathology, except for age (Fig. 2a,c). Specifically, the accuracy was significantly lower in participants aged between 73 and 80 years (87%, 95% CI = 85–90%, *P* = 0.046) and aged ≥80 years (83%, 95% CI = 79–88%, *P* = 0.003) compared with those aged <73 years (91%, 95% CI = 88–92%). A numerically lower accuracy in older participants was observed across all cohorts, except for the Brescia cohort (Supplementary Table 2). The significant effect of age on accuracy persisted even after excluding participants with CKD, which is known to be more common in older individuals (Supplementary Table 2). When applying the two-cutoff approach, no significant effect of age or any other demographic factor or comorbidity was observed (Fig. 2b). However, in older individuals and those with CKD, the percentage of individuals with intermediate p-tau217 results increased to 19–20% (Fig. 2d). Examining the effects of older age and comorbidities in primary care, where they were more prevalent, revealed no significant effect on accuracy (Extended Data Fig. 3).

### Accuracy across disease stages

**Fig. 3:**
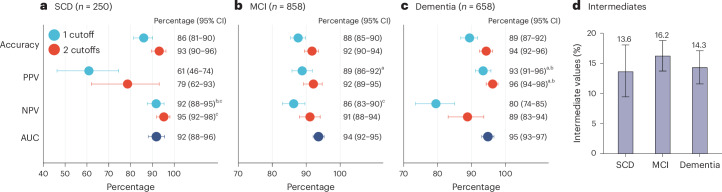
Performance of plasma p-tau217 (Lumipulse) across cognitive stages. **a**–**c**, Using pooled data from all five cohorts (*n* = 1767), performance shown for participants with SCD (*n* = 250) (**a**), MCI (*n* = 858) (**b**) and dementia (*n* = 658) (**c**). Results are shown using a single cutoff (blue) or two cutoffs (red). Note that AUC values are independent of cutoffs. **d**, Participants who fell between the two cutoffs classified as intermediate. Data are presented as the observed percentage and the error bars as the 95% CI derived from the bootstrap distribution. AD pathology was defined as CSF Aβ42:p-tau181 < 11.94 or positive visual read on amyloid PET if lumbar puncture was not performed. To assess whether the observed difference in the statistics is significantly different from zero, we performed a bootstrap hypothesis test. The *P* value (two sided) was calculated as the proportion of bootstrap resamples (*n* = 2,000) where the absolute null-distributed statistic was greater than or equal to the observed difference. Differences between AUCs were assessed using DeLong statistics. Results were not corrected for multiple comparisons. Significant *P* values in the order as presented in the plot: 0.040, 0.001, 0.038 (**a**); <0.001, 0.046 (**b**); <0.001, 0.017, 0.024, 0.016 (**c**). ^a^Significantly higher than the SCD group, *P* < 0.05. ^b^Significantly higher than the MCI group, *P* < 0.05. ^c^Significantly higher than the dementia group, *P* < 0.05.

When comparing Lumipulse p-tau217 accuracies and AUCs across groups with subjective cognitive decline (SCD), mild cognitive impairment (MCI) and dementia, no significant differences were found. Accuracies ranged between 86% and 89% using one cutoff and 92% and 94% using two cutoffs (Fig. 3a–c). As expected, based on the underlying prevalence of AD pathology, the NPV was higher in the SCD group whereas the PPV was higher in the dementia group. No significant differences in the percentage of intermediate results were found (Fig. 3d).

### Comparison of plasma p-tau217 and the p-tau217:Aβ42 ratio

**Fig. 4:**
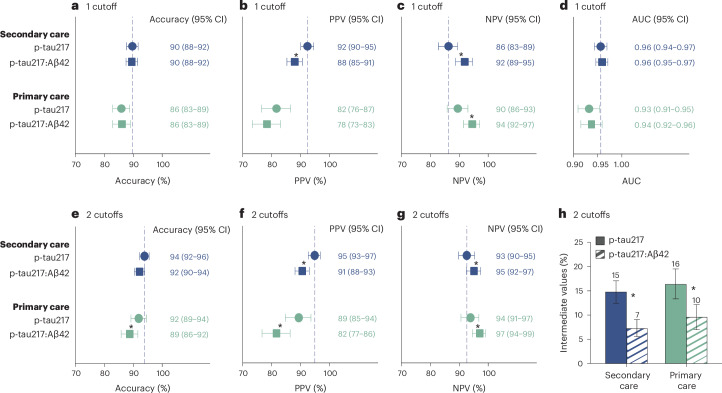
Comparison between plasma p-tau217 and p-tau217:Aβ42 (Lumipulse) for discriminating AD pathology-positive versus AD pathology-negative participants. **a**–**c**,**e**–**h**, Pooled data from the secondary care cohorts (*n* = 911) and the primary care cohort (*n* = 502) examined. In the single cutoff approach (**a**–**c**). The cutoffs were >0.27 pg ml^−1^ for p-tau217 and >0.008 pg ml^−1^ for p-tau217:Aβ42. In the two-cutoff approach, the cutoffs for p-tau217 were <0.22 pg ml^−1^ and >0.34 pg ml^−1^ and <0.007 pg ml^−1^ and >0.009 pg ml^−1^ for p-tau217:Aβ42 (**e**–**h**). **d**, Note that the AUC values are independent of cutoffs. Cutoffs were established in the Malmö secondary care cohort (*n* = 337). Data are presented as the observed percentage and the error bars as the 95% CI derived from the bootstrap distribution. AD pathology was defined as CSF Aβ42:p-tau181 < 11.94 or positive visual read on amyloid PET if lumbar puncture was not performed. To assess whether the observed difference in the statistics is significantly different from zero, we performed a bootstrap hypothesis test. The *P* value (two sided) was calculated as the proportion of bootstrap resamples (*n* = 2,000) where the absolute null-distributed statistic was greater than or equal to the observed difference. Differences between AUCs were assessed using DeLong statistics. Results were not corrected for multiple comparisons. Significant *P* values in the order as presented in the plot: <0.001 (**b**); <0.001, 0.001 (**c**); 0.014 (**e**); <0.001, <0.001 (**f**); 0.007, 0.010 (**g**); <0.001, < 0.001 (**h**). ^*^Significant difference between the two biomarkers (*P* < 0.05).

**Extended Data Fig. 4:**
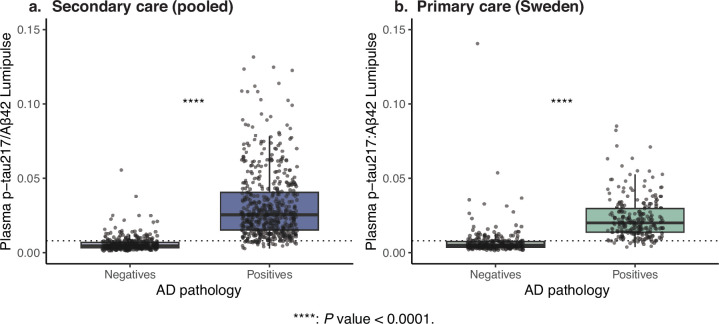
Boxplots of plasma p-tau217/Aβ42 (Lumipulse) concentrations with AD pathology as grouping variable. Boxplots of plasma p-tau217/Aβ42 (Lumipulse) concentrations in the (**a**) pooled secondary care cohort (*n* = 911) and (**b**) primary care cohort (*n* = 502) with AD pathology as grouping variable. Plasma p-tau217/Aβ42 differed significantly ( *P* < 0.001) between AD positive and AD negative participants in both populations. Dashed lines represent the single cutoff at 90% specificity established in the Malmö secondary care cohort. The dots represent individual participants. The central band of the boxplot represents the group median, the box limits correspond to the first and third quartiles, and the whiskers represent the minimum/maximum value or the 1.5 interquartile range (IQR) from the box, whichever is smaller. AD pathology was defined as CSF Aβ42/p-tau181 < 11.94 or positive amyloid PET visual read for those who did not undergo lumbar puncture. Group mean levels were compared with Student’s t-test. N = 10 outliers are not shown to improve visualization, but individuals were used in the analyses. One outlier (0.156) was AD pathology-negative. The remaining 9 outliers corresponded to AD pathology-positive participants, with the following plasma p-tau217/Aβ42 levels: 0.223, 0.256, 0.260, 0.273, 0.287, 0.509, 0.671, 0.761, 0.872. All outliers had MCI or dementia. Abbreviations: AD, Alzheimer’s disease; CKD, chronic kidney disease; IQR, interquartile range.

Plasma p-tau217:Aβ42 (Lumipulse) was available for 502 primary care participants and 911 secondary care participants (Supplementary Table 3). The p-tau217:Aβ42 levels were significantly higher in AD pathology-positive versus AD pathology-negative participants (Extended Data Fig. 4). The p-tau217:Aβ42 ratio provided similar accuracy and AUC compared with p-tau217 in primary and secondary care using a single cutoff (Fig. 4a–d). Using the two-cutoff approach, the accuracy of p-tau217:Aβ42 was significantly worse in primary care (Fig. 4e–g; *P* = 0.014). However, p-tau217:Aβ42 reduced the number of participants with intermediate results in both primary and secondary care from 15–16% to 7–10% (Fig. 4h; both *P* < 0.001). In addition, p-tau217:Aβ42 had a lower PPV and higher NPV in both primary and secondary care.

**Extended Data Fig. 5:**
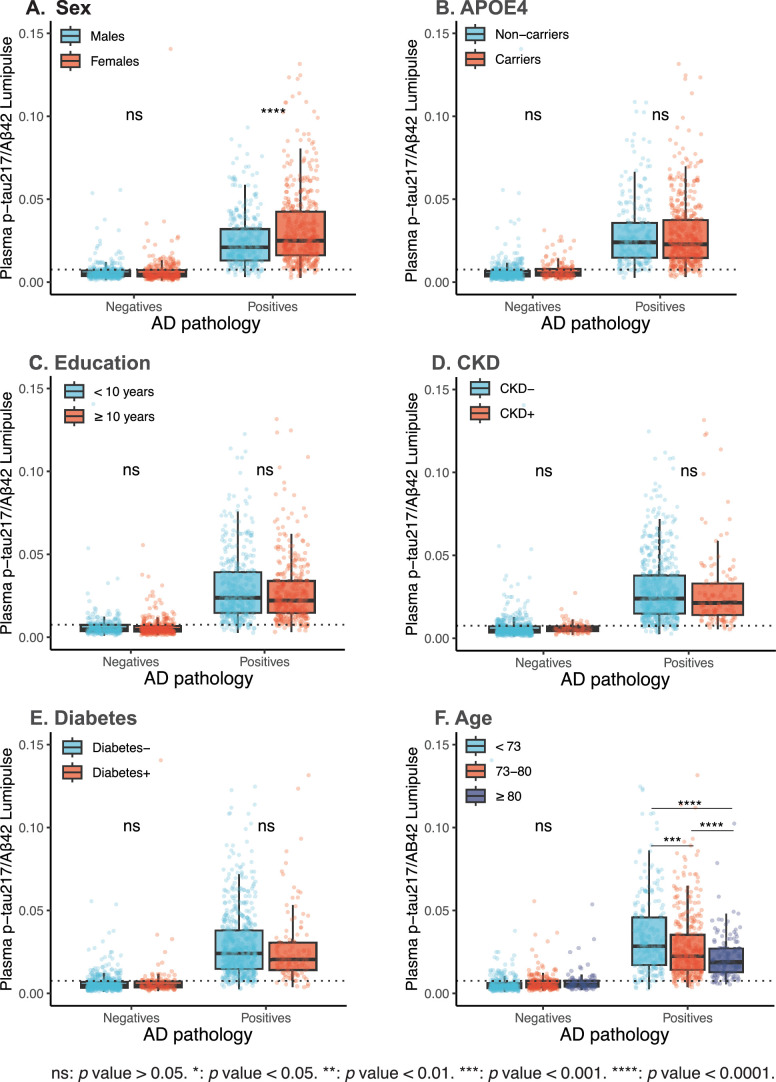
Plasma p-tau217/Aβ42 (Lumipulse) and the effect of demographic characteristics and comorbidities. Boxplots of plasma p-tau217/Aβ42 concentrations stratified by AD pathology and the following variables: (**a**) sex, (**b**) *APOE* ε4 carriership, (**c**) education, (**d**), chronic kidney dysfunction, (**e**) presence of diabetes, and (**f**) age. The analyses were performed pooling the plasma p-tau217/Aβ42 data of the five cohorts (*n* = 1413). Dashed lines represent the single cutoff at 90% specificity established in the Malmö secondary care cohort. The dots represent individual participants. The central band of the boxplot represents the group median, the box limits correspond to the first and third quartiles, and the whiskers represent the minimum/maximum value or the 1.5 interquartile range (IQR) from the box, whichever is smaller. AD pathology was defined as CSF Aβ42/p-tau181 < 11.94 or positive amyloid PET visual read for those who did not undergo lumbar puncture. Group mean levels were compared with Student’s t-test. N = 10 outliers are not shown to improve visualization, but individuals were used in the analyses. One outlier (0.156) was AD pathology-negative. The remaining 9 outliers corresponded to AD pathology-positive participants, with the following plasma p-tau217/Aβ42 levels: 0.223, 0.256, 0.260, 0.273, 0.287, 0.509, 0.671, 0.761, 0.872. All outliers had MCI or dementia. Abbreviations: AD, Alzheimer’s disease; CKD, chronic kidney disease; IQR, interquartile range.

In contrast to plasma p-tau217, no increase in plasma p-tau217:Aβ42 ratio was observed in individuals with CKD regardless of AD pathology status or in older individuals without AD pathology (Extended Data Fig. 5). Similar to p-tau217, p-tau217:Aβ42 levels were increased in women and in younger participants with AD pathology status (Extended Data Fig. 5).

### Comparison of Lumipulse and MS-based assays

**Fig. 5:**
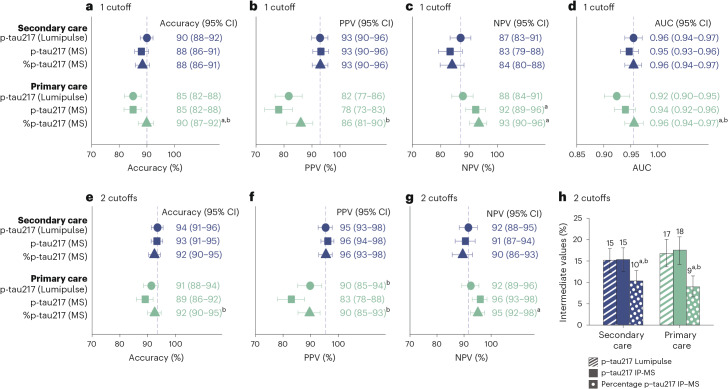
Comparisons between plasma Lumipulse p-tau217 and MS-based p-tau217 and %p-tau217 for discriminating AD pathology-positive versus AD pathology-negative participants. Data from the pooled secondary care cohorts (*n* = 619) and the primary care cohort (*n* = 513) were examined. The secondary care cohorts consisted of participants from the Malmö *(n* = 337), Gothenburg (*n* = 164) or Brescia (*n* = 118) cohort with MS-based data available. Cutoffs were set in the Malmö secondary care cohort (*n* = 337). **a**–**c**, In the single cutoff approach, the cutoffs were >0.27 pg ml^−1^ for Lumipulse p-tau217, >2.27 pg ml^−1^ for MS-based p-tau217 and >4.27 pg ml^−1^ for MS-based %p-tau217. **e**–**h**, In the two-cutoff approach, the cutoffs for Lumipulse p-tau217 were <0.22 pg ml^−1^ and >0.34 pg ml^−1^, <1.59 pg ml^−1^ and >2.92 pg ml^−1^ for MS-based p-tau217 and <3.55 pg ml^−1^ and >5.08 pg ml^−1^ for MS-based %p-tau217. Data are presented as the observed percentage and the error bars as the 95% CI derived from the bootstrap distribution. AD pathology was defined as CSF Aβ42:p-tau181 < 11.94 or positive visual read on amyloid PET if lumbar puncture was not performed. To assess whether the observed difference in the statistics is significantly different from zero, we performed a bootstrap hypothesis test. The *P* value (two sided) was calculated as the proportion of bootstrap resamples (*n* = 2,000) where the absolute null-distributed statistic was greater than or equal to the observed difference. Differences between AUCs were assessed using DeLong statistics. Results were not corrected for multiple comparisons. Significant *P* values in the order as presented in the plot: 0.003, <0.001 (**a**); <0.001 (**b**); 0.020, 0.004, <0.001 (**c**); 0.006, 0.025 (**d**); 0.020 (**e**); 0.008, 0.006 (**f**); 0.014 (**g**); 0.003, <0.001, <0.001, <0.001 (**h**). ^a^Significantly better than Lumipulse p-tau217, *P* < 0.05. ^b^Significantly better than MS-based p-tau217, *P* < 0.05.

**Extended Data Fig. 6:**
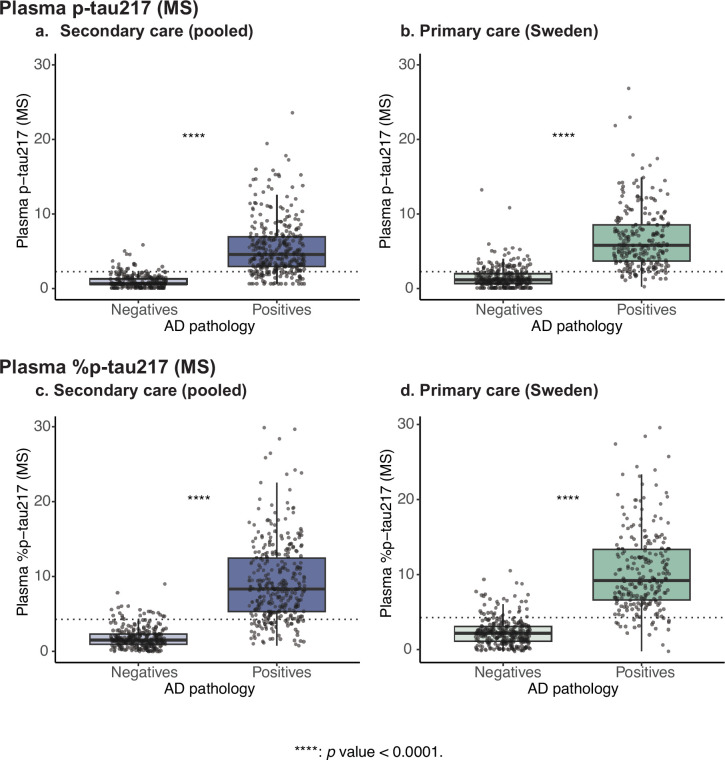
Boxplots of mass spectrometry-based plasma p-tau217 and %p-tau217 with AD pathology as grouping variable. Pooled secondary care cohort data (**a**, **c**, *n* = 619) and primary care data (**b**, **d**, *n* = 513) with AD pathology as grouping variable to assess differences in MS-based p-tau217 (**a** and **b**) and %p-tau217 (**c** and **d**). Plasma levels differed significantly (*P* < 0.0001) between AD pathology-positive and AD pathology-negative participants in both populations. Dashed lines represent the single cutoff at 90% specificity established in the Malmö secondary care cohort. The dots represent individual participants. The central band of the boxplot represents the group median, the box limits correspond to the first and third quartiles, and the whiskers represent the minimum/maximum value or the 1.5 interquartile range (IQR) from the box, whichever is smaller. AD pathology was defined as CSF Aβ42/p-tau181 < 11.94 or positive amyloid PET visual read for those who did not undergo lumbar puncture. Group mean levels were compared with Student’s t-test. Abbreviations: AD, Alzheimer’s disease; MS, mass spectrometry; IQR, interquartile range.

MS-based p-tau217 and %p-tau217 data were available in a subset of patients from the Malmö (*n* = 337), Gothenburg (*n* = 164) and Brescia (*n* = 118) secondary care cohorts, as well as the primary care cohort (*n* = 513) (Supplementary Table 4 and Extended Data Fig. 6). No significant differences in AUCs or accuracies were found between the Lumipulse p-tau217 and MS-based p-tau217 or %p-tau217 assays in secondary care using either the single or the two-cutoff approaches (Fig. 5). In the primary care cohort, MS-based %p-tau217 had a significantly higher accuracy (90%, 95% CI = 87–92) and AUC (0.96, 95% CI = 0.94–0.97) than Lumipulse p-tau217 (accuracy = 85%, 95% CI = 82–88, *P* = 0.003; AUC = 0.92, 95% CI = 0.90–0.95, *P* = 0.006) and MS-based p-tau217 (accuracy = 85%, 95% CI = 82–88%, *P* < 0.001; AUC = 0.94, 95% CI = 0.92–0.96, *P* = 0.025) when using the single cutoff approach. The accuracy of Lumipulse p-tau217 in primary care was not statistically different from MS-based p-tau217 or %p-tau217 when using two cutoffs, but resulted in a significantly higher proportion of participants with intermediate results (Fig. 5h; all *P* < 0.003).

### Impact of patient characteristics and stage on assay accuracy

**Extended Data Fig. 7:**
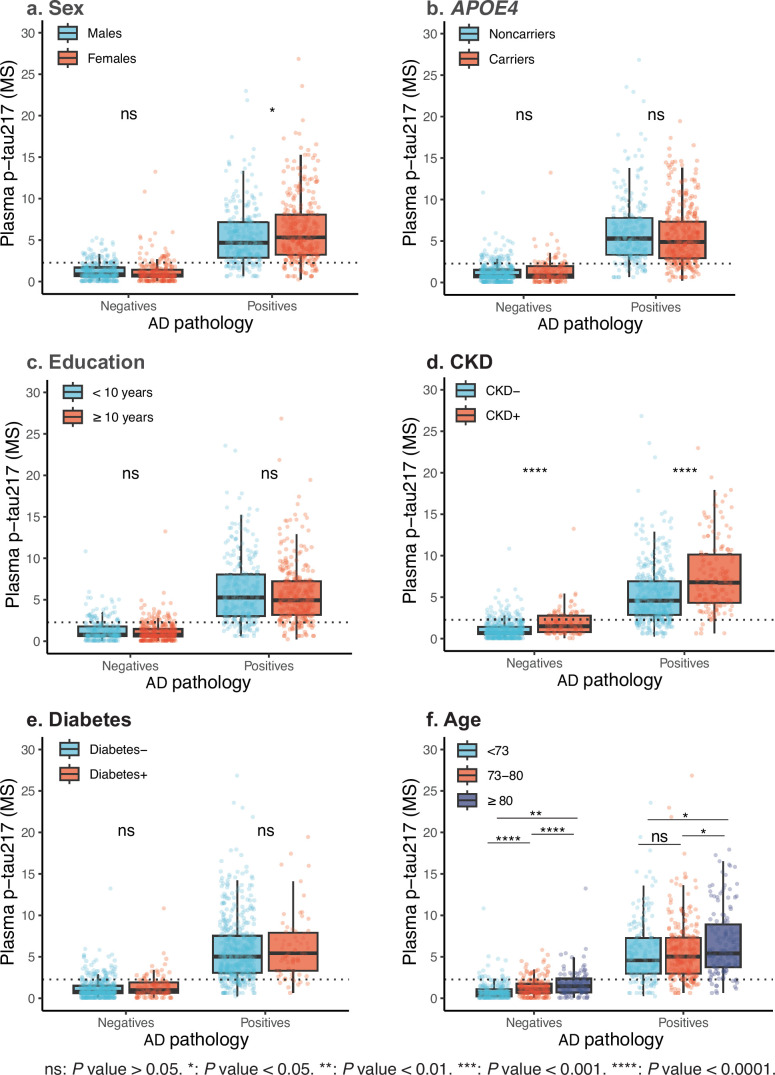
Mass spectrometry-based plasma p-tau217 and the effect of demographic characteristics and comorbidities. Concentrations stratified by AD pathology and the following variables: (**a**) sex, (**b**) *APOE* ε4 carriership, (**c**) education, (**d**), chronic kidney dysfunction, (**e**) presence of diabetes, and (**f**) age. The analyses were performed pooling the data of the four cohorts with MS data available (*n* = 1132). Dashed lines represent the single cutoff at 90% specificity established in the Malmö secondary care cohort. The dots represent individual participants. The central band of the boxplot represents the group median, the box limits correspond to the first and third quartiles, and the whiskers represent the minimum/maximum value or the 1.5 interquartile range (IQR) from the box, whichever is smaller. AD pathology was defined as CSF Aβ42/p-tau181 < 11.94 or positive amyloid PET visual read for those who did not undergo lumbar puncture. Group mean levels were compared with Student’s t-test. Abbreviations: AD, Alzheimer’s disease; CKD, chronic kidney disease; MS, mass spectrometry; IQR, interquartile range.

**Extended Data Fig. 8:**
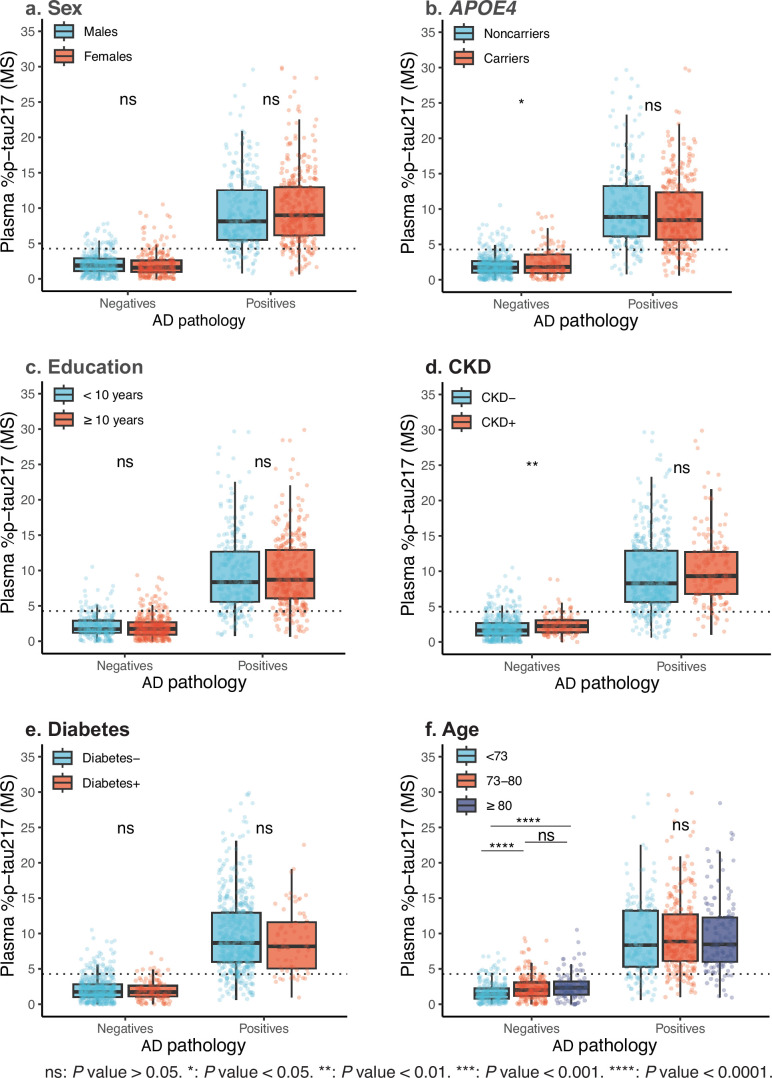
Mass spectrometry-based plasma %p-tau217 and the effect of demographic characteristics and comorbidities. Plasma %p-tau217 stratified by AD pathology and the following variables: (**a**) sex, (**b**) *APOE* ε4 carriership, (**c**) education, (**d**), chronic kidney dysfunction, (**e**) presence of diabetes, and (**f**) age. The analyses were performed pooling the data of the four cohorts with MS data available (*n* = 1132). Dashed lines represent the single cutoff at 90% specificity established in the Malmö secondary care cohort. The dots represent individual participants. The central band of the boxplot represents the group median, the box limits correspond tothe first and third quartiles, and the whiskers represent the minimum/maximum value or the 1.5 interquartile range (IQR) from the box, whichever is smaller. AD pathology was defined as CSF Aβ42/p-tau181 < 11.94 or positive amyloid PET visual read for those who did not undergo lumbar puncture. Group mean levels were compared with Student’s t-test. Abbreviations: AD, Alzheimer’s disease; CKD, chronic kidney disease; MS, mass spectrometry; IQR, interquartile range.

**Extended Data Fig. 9:**
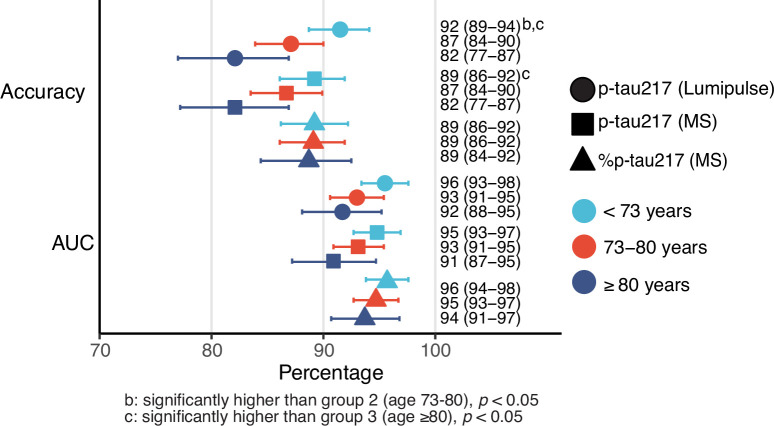
Comparison of assay performances between different age groups. This figure presents the accuracy and predictive values of plasma p-tau217 (Lumipulse) and p-tau217 (MS) and %p-tau217 (MS) across three age groups to examine the impact of demographic characteristics and comorbidities. The analysis combined data from four different cohorts with MS data available (*n* = 1132). Results are shown using a single cutoff. The dots represent the actual percentage, and the error bars the 95% CI derived from the bootstrap distribution. AD pathology was defined as CSF Aβ42/p-tau181 < 11.94 or positive visual read on amyloid PET if lumbar puncture was not performed. To assess whether the observed difference in the statistics is significantly different from zero, we performed a bootstrap hypothesis test. The *P*value (two-sided) was calculated as the proportion of bootstrap resamples (n = 2000) where the absolute null-distributed statistic was greater than or equal to the observed difference. Differences between AUCs were assessed with DeLong statistics. Results were not corrected for multiple comparisons. The comparisons between age groups per biomarker are shown in the figure (*P* values in the order as presented in the plot: 0.012; <0.001; 0.013). %p-tau217 (MS) had significantly higher AUCs than p-tau217 (MS) when comparing the age categories 73-80 (*P* = 0.033) and ≥80 (*P* = 0.013). No differences were observed between the biomarkers within the different age categories. Abbreviations: Accuracy, percent correctly classified participants; CI, confidence interval; MS: mass spectrometry; NPV, negative predictive value; PPV, positive predictive value.

Among AD pathology-positive participants, %p-tau217 levels did not differ between any of the demographic or comorbidity groups. Specifically, the lower levels of Lumipulse p-tau217 and p-tau217:Aβ42 observed in the older age groups was not observed with MS-based p-tau217 and %p-tau217 (Extended Data Figs. 7 and 8). As age was the only demographic variable that showed differences in accuracy for Lumipulse p-tau217, we compared the accuracy and AUC of Lumipulse p-tau217 with MS-based p-tau217 and %p-tau217 across the three different age groups (Extended Data Fig. 9). As observed earlier, Lumipulse p-tau217 demonstrated lower accuracy in the older participants in this subsample (all *P* < 0.012), whereas no differences between age categories were observed for MS-based %p-tau217 (89% in all age groups).

**Extended Data Fig. 10:**
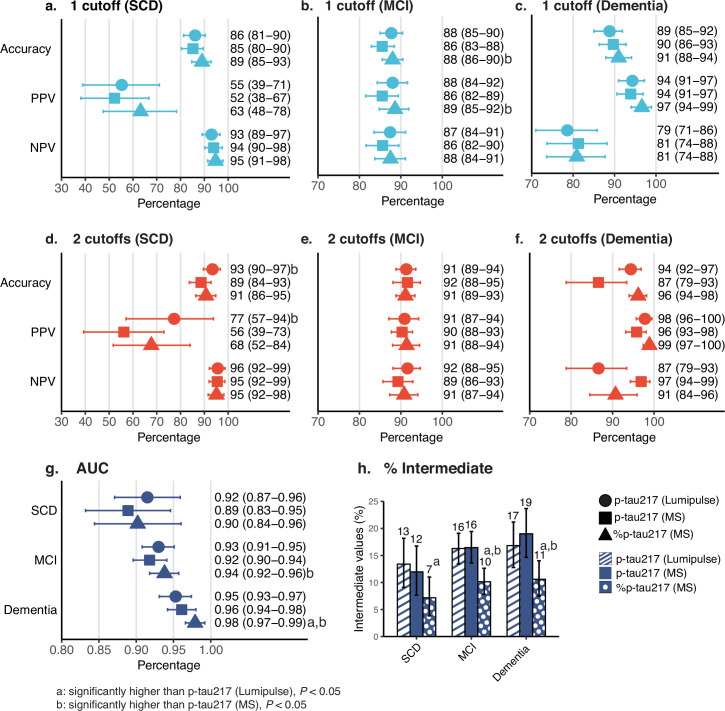
Performances of plasma p-tau217 (Lumipulse) vs. mass spectrometry-based plasma p-tau217 and %p-tau217 across disease stages. This figure presents the accuracy and predictive values of plasma p-tau217 (Lumipulse) and plasma p-tau217 (MS) and %p-tau217 (MS) across the disease stages SCD (**a**), MCI (**b**) and dementia (**c**). Results are shown using a single cutoff (blue, **a**-**c**) and two cutoffs (red, SCD (**d**), MCI (**e**) and dementia (**f**). Note that AUC values are independent of cutoffs (**g**). Participants who fall between the two cutoffs were classified in the intermediate group (**h**). The dots or bars represent the actual percentage, and the error bars the 95% CI. The analysis combined data from four different cohorts with MS data available (*n* = 1132). AD pathology was defined as CSF Aβ42/p-tau181 < 11.94 or positive visual read on amyloid PET if lumbar puncture was not performed. To assess whether the observed difference in the statistics is significantly different from zero, we performed a bootstrap hypothesis test. The *P* value (two-sided) was calculated as the proportion of bootstrap resamples (n = 2000) where the absolute null-distributed statistic was greater than or equal to the observed difference. Differences between AUCs were assessed with DeLong statistics. Results were not corrected for multiple comparisons. Significant *P* values in the order as presented in the plot: (**b**) 0.043; 0.046, (**d**) 0.033; 0.024, (**g**) 0.003; 0.009; 0.006, (**h**) 0.027; <0.001; <0.001; 0.005; <0.001. Abbreviations: CI, confidence interval; MS: mass spectrometry; MCI, mild cognitive impairment; NPV, negative predictive value; PPV, positive predictive value; SCD, subjective cognitive decline.

There were no significant differences in accuracy, PPVs or NPVs between p-tau217 measured by Lumipulse and MS-based %p-tau217 across the three cognitive stages (SCD, MCI and dementia), regardless of whether one or two cutoffs were used (Extended Data Fig. 10). However, in the dementia group, %p-tau217 measured by MS demonstrated a significantly higher AUC compared with Lumipulse p-tau217 (*P* = 0.009). Furthermore, across all three cognitive groups, %p-tau217 classified significantly fewer individuals into the intermediate zone than Lumipulse p-tau217 (all *P* < 0.027).

### Cost-effectiveness analysis of plasma p-tau217 (Lumipulse)

A cost-effectiveness analysis was performed comparing the Lumipulse p-tau217 assay with CSF analysis and amyloid PET imaging (Supplementary Table 5) and is available at https://bbrc-lab.shinyapps.io/Cost-effectiveness_analysis_plasma_p-Tau217. Using approximated costs for CSF AD biomarkers and amyloid PET imaging in the United States of America, we estimated savings of up to 60% compared with CSF testing alone and up to 81% compared with amyloid PET imaging alone, when implementing a two-cutoff approach with Lumipulse p-tau217. This calculation assumes that 14.7% of patients fall into the intermediate group (the total proportion in the present study) and require further testing (Supplementary Table 5). The app allows users to input region-specific costs for CSF and PET, enabling tailored cost analyses. A screenshot of the app is shown in Supplementary Fig. 1.

### Sensitivity analyses

We performed additional analyses to assess the performance of Lumipulse p-tau217 and confirm the robustness of our results. First, the CSF Aβ42:40 ratio was used as a reference standard, instead of CSF Aβ42:p-tau181, with the FDA-approved cutoff of ≤0.072 pg ml^−1^ for positivity (Supplementary Fig. 2)^12^. Second, analyses were performed in the Swedish primary care cohort using only the CSF Aβ42:p-tau181 outcome, excluding those for whom Aβ pathology was defined by amyloid PET *(n* = 87; Supplementary Fig. 3). Third, a single cutoff established using the highest Youden index for AD pathology was used, instead of defining it at 90% specificity (Supplementary Fig. 4). The results of these sensitivity analyses were very similar to the main analysis.

## Discussion

We found that the fully automated Lumipulse assay measuring plasma p-tau217 has a high accuracy in differentiating patients with AD pathology from those without. Several noteworthy aspects of the present study merit emphasis. First, the cutoffs were established in an independent cohort and then crossvalidated in several cohorts. Unlike many biomarker studies that develop ad-hoc plasma cutoffs specific to their cohort, our approach supports generalizability, facilitating broader use of the biomarker. This is of particular importance for tests intended for use in clinical practice using predefined cutoffs. Second, the high performance of the assay was consistent across four out-of-sample cohorts from different countries (Fig. 1), which included populations representing patients seeking advice for cognitive symptoms in specialized memory clinics and primary care. Moreover, the analyses of the plasma samples were performed in different centers and not centralized in one single laboratory. Third, we demonstrated that the accuracy was similar across different demographic, comorbidity and cognitive stage groups, except for a lower accuracy in older individuals, where a two-cutoff approach was necessary to achieve comparable accuracy (Figs. 2 and 3). Finally, we demonstrated, in a subset of patients, that the fully automated p-tau217 assay exhibits a performance comparable to MS-based measurements of p-tau217 in secondary care and slightly lower performance in primary care and older individuals, but not when using the two-cutoff approach (Fig. 5 and Extended Data Fig. 9). Given the suggested requirement of ≥90% accuracy for BBMs in the diagnosis of AD^15^, a two-cutoff approach for the Lumipulse p-tau217 assay might be necessary for its use as a stand-alone test for AD pathology, especially in primary care and across different age groups and cognitive stages (Figs. 1–3).

Although it has been known for several years that some BBMs can accurately detect AD pathology, these assays have not been widely available in most clinical centers and have been primarily limited to research cohorts. Therefore, the development of fully automated, user-friendly and reproducible tests is essential for this technology to become accessible to most centers. Lumipulse plasma assays have not previously been validated with predefined cutoffs and have been examined only in single-center studies^16–18^. A recent US-based study established a cutoff for p-tau217 at 0.25 pg ml^−1^ using the 90% specificity approach that we applied, which is comparable to the 0.27-pg ml^−1^ cutoff in our study^17^. For p-tau217:Aβ42, they only reported a cutoff at 85% specificity (0.008), which still aligns with our finding at 90% specificity (also 0.008). Similar to our study, they found that the p-tau217:Aβ42 ratio did not improve the accuracy but reduced the proportion with intermediate results. These results further support the generalizability of our cutoffs and results.

Comorbidities, especially CKD, are known to impact BBMs^19,20^. Notably, in our study the accuracy of Lumipulse p-tau217 was not affected by CKD. However, the presence of CKD resulted in a higher proportion of participants being classified into the intermediate group when two cutoffs were used (Fig. 2), presumably by the slight increase in p-tau217 concentration in participants without AD pathology (Extended Data Fig. 2d). This issue was mitigated by using the plasma p-tau217:Aβ42 ratio, with no increased levels observed among individuals with CKD (Extended Data Fig. 5d), consistent with previous findings for plasma ratios^20^. Another potential advantage of the plasma p-tau217:Aβ42 ratio was its higher NPV which could be especially important for primary care, although this comes at the cost of a lower PPV (Fig. 4). This phenomenon was observed in both primary and secondary care, and the only previous study reporting on this ratio observed a similar result using a Youden index cutoff for plasma p-tau217:Aβ42 (ref. ^17^).

The performance of Lumipulse p-tau217 significantly decreased with age (Fig. 2). This decline was not attributable to any specific cohort (Supplementary Table 2), including the primary care cohort (Extended Data Fig. 3), which had older participants and showed lower accuracy for Lumipulse p-tau217. The lower accuracy in the older individuals could potentially be explained by the lower p-tau217 concentration observed in the older participants with AD pathology, in combination with a higher p-tau217 concentration in older participants without AD pathology (Extended Data Figs. 2 and 5; lower levels in AD pathology-positive individuals were also observed for p-tau217:Aβ42). This could also explain the higher proportion of intermediate Lumipulse p-tau217 results in older participants when the two-cutoff approach was used (Fig. 2d). Previous studies have demonstrated that older individuals with AD presenting with cognitive symptoms in memory clinics often exhibit lower amounts of AD pathology, particularly tau pathology, partly as a result of the presence of other brain pathologies and lower cognitive reserve^21^. It is interesting that this age-related effect in AD pathology was not observed for %p-tau217 results, which showed consistent levels across age groups (Extended Data Fig. 8) and no age-related impact on accuracy (Extended Data Fig. 9). Other factors underlying this finding should be explored in future studies. Additional differences between Lumipulse p-tau217 and %p-tau217 included less influence of sex and CKD on %p-tau217, again highlighting the potential robustness provided by using a ratio (compare Extended Data Figs. 2, 7 and 8).

A key advantage of using BBMs is the potential to reduce the need for lumbar punctures, CSF analyses and amyloid PET scans, leading to substantial cost savings. This is especially timely because new disease-modifying drugs are becoming available in some countries, requiring biomarker confirmation of AD pathology. Given the considerable variation in costs for these tests across countries and centers, we created an online application to calculate the cost savings of using plasma p-tau217 (Lumipulse) compared with the local costs of CSF AD core biomarkers or amyloid PET (Supplementary Fig. 1). For example, using the approximate costs of CSF AD core biomarkers or amyloid PET in the United States of America, we estimated savings of up to 60% compared with solely using CSF tests and up to 81% compared with amyloid PET, using a two-cutoff approach and assuming that 14.7% of patients fall into the intermediate zone, which was the pooled proportion of intermediate results in the present study (Supplementary Table 5).

The present study has some limitations. First, our study included only European cohorts, necessitating further validation in clinical cohorts from other regions worldwide. Second, the slightly lower performance compared with %p-tau217 in primary care settings and among older age groups highlights the need for further investigation.

In summary, the utilization of a fully automated assay to measure plasma p-tau217, employing pre-established cutoffs, demonstrates high accuracy in detecting AD pathology across various specialized memory clinics and in primary care. These promising findings, coupled with the feasibility of implementing this technique in different settings, might facilitate the adoption of a BBM into routine clinical practice for more accurate AD diagnostics.

## Methods

### Participants

All participants were recruited as part of undergoing a memory investigation in clinical practice to ensure a representative, real-life study population. They all provided written informed consent. The studies were approved by the Swedish Ethical Review Authority (the Malmö, Gothenburg and primary care cohorts), the independent ethics committee, ‘Parc de Salut Mar’ Barcelona, Spain (the Barcelona cohort) and the Spedali Civili of Brescia local ethics committee, Italy (the Brescia cohort). The inclusion criteria for each cohort are described below.

In the BioFINDER-Memory Clinic study (NCT06122415)^9^, termed the Malmö cohort, the inclusion criteria were: (1) being under investigation for cognitive symptoms at the Memory Clinic of Skåne University Hospital, Sweden; and (2) CSF and blood sampling planned to be done as part of clinical practice even if the patient was not taking part in the present study. The exclusion criteria were: (1) not undergoing CSF or blood sampling as part of clinical practice and (2) not undergoing cognitive testing as part of clinical practice. Patients included in the present study were consecutively recruited between December 2022 and November 2023.

In the H70 Clinical Studies (termed the Gothenburg cohort), the inclusion criterion will be under investigation for cognitive symptoms at the Memory Clinic of Sahlgrenska University Hospital, Sweden. There were no exclusion criteria. For the present study, participants with CSF and blood sampling as part of the clinical investigation were included. Patients in the present study were consecutively recruited between March 2020 and June 2023.

In the BIODEGMAR study^3^ (termed the Barcelona cohort), the inclusion criteria were: (1) undergoing evaluation at the Cognitive and Behavioural Neurology Unit and inclusion in the DEGMAR register; (2) signed informed consent; and (3) having one of the following clinical diagnoses: SCD, MCI, AD dementia; behavioral variant frontotemporal dementia; progressive aphasia or primary progressive aphasia (logopenic, nonfluent and semantic variants); Lewy body dementia; corticobasal syndrome; progressive supranuclear palsy syndrome; and vascular cognitive impairment and dementia. Individuals with other causes of dementia, but unspecified clinical diagnoses, were also included and categorized as ‘other’. The exclusion criteria were: (1) age ≥80 years; (2) contraindication for lumbar puncture; or (3) disagreement with study procedures. Patients included in the present study were consecutively recruited between April 2017 and November 2023.

In the Life-BIO cohort (termed the Brescia cohort), the inclusion criteria were participants with MCI or mild dementia who underwent clinical routine CSF assessment at the outpatient neurodegenerative clinic of the Brescia University Hospital, Italy. The following exclusion criteria were applied: (1) cortical or subcortical cerebrovascular infarcts in structural imaging; (2) other neurological disorders or medical conditions potentially associated with cognitive deficits; (3) bipolar disorder, schizophrenia, history of drug or alcohol abuse or impulse control disorder; (4) recent traumatic events or acute fever or inflammation; and (5) refusal of collection of blood sampling for research purposes. Patients in this study were consecutively recruited between March 2020 and November 2023.

In the BioFINDER-Primary Care study (NCT06120361)^9^ (termed primary care cohort), the inclusion criteria were: (1) patient seeks medical help in primary care because of cognitive symptoms experienced by the patient or informant, or the primary care physician suspects a neurodegenerative disorder; (2) age ≥40 years; and (3) cognitive impairment characterized as SCD, MCI or mild dementia. The exclusion criteria were: (1) already diagnosed dementia; (2) substantial unstable systemic illness making it difficult to participate in the study; (3) current substantial alcohol or substance misuse; (4) refusing investigation at the memory clinic; (5) cognitive impairment with acute onset as a result of a stroke; and (6) cognitive impairment that can, with high certainty, be assessed by the primary care physician or explained by another condition or disease such as psychotic disorder, depression or alcohol abuse. Patients included in the present study were consecutively recruited from 19 primary care units in the south of Sweden from January 2020 to November 2023.

### Criteria for SCD, MCI and dementia

In each cohort, syndromic diagnosis of SCD, MCI or dementia was done using established criteria^22–24^ or the Clinical Dementia Rating (CDR) scale^25^.

In the Malmö cohort^9^, SCD was defined as experiencing cognitive symptoms to the level that the patient was referred to the memory clinic owing to cognitive symptoms, but not fulfilling the criteria for MCI or dementia. MCI was defined as having cognitive symptoms and exhibiting objective cognitive impairment in any cognitive domain, based on the following neuropsychological battery: Trail Making Test A, Trail Making Test B and symbol digit modalities test (attention or executive function); verbal fluency animals, letter S fluency and the 15-word short version of the Boston naming test (verbal function); ten-word immediate and delayed recall from the AD assessment scale (ADAS), as well as a recognition task of the ten words (memory); and incomplete letters and cube analysis from the visual object and space perception battery (VOSP; visuospatial function). Objective cognitive impairment was not defined according to a strict threshold but was based on the overall clinical assessment, taking into account premorbid function. Dementia was diagnosed according to the *Diagnostic and Statistical Manual of Mental Disorders*, 5th edn (DSM-5) criteria for major neurocognitive disorder^26^.

In the Gothenburg cohort, SCD was defined as having cognitive symptoms and global CDR = 0, MCI as global CDR = 0.5 and dementia as global CDR ≥ 1 (ref. ^25^). CDR scoring was performed by experienced clinical staff at the memory clinic (physicians, nurses or neuropsychologists), based on routine examinations, patient interviews, reports from close relatives, cognitive tests (Mini-Mental State Examination (MMSE), Montréal Cognitive Assessment (MoCA) and the symbol digit modalities test), Lawton–Brody Instrumental Activities of Daily Living Scale (IADL)^27^, the Functional Activities Questionnaire^28^ and, in some cases, also neuropsychological testing (the Boston Naming Test, Processing Speed Identical Forms, Rey Auditory Verbal Learning Test (RAVLT), VOSP, Trail Making Test A and B, number repetition test, letter fluency, animal fluency test and Stroop and block design test).

In the Barcelona cohort, SCD was defined as subjective complaints of cognitive decline with normal performance on neuropsychological testing adjusted for age and educational level^22^. MCI was defined as clinical symptoms suggestive of cognitive decline with objective cognitive impairment in at least one cognitive domain on formal neuropsychological evaluation, with minor changes in ADLs, not meeting the criteria of dementia, and a global CDR score of 0.5. Dementia was defined as cognitive decline with objective impairment in at least one cognitive domain on neuropsychological evaluation and a CDR score of ≥1. The neuropsychological evaluation in the Barcelona cohort included the following cognitive tests and functional scales: MMSE, Memory Impairment Screen, Automatic reverse series (subtest of test Barcelona cognitive battery), semantic and phonetic fluency tasks (subtest of test Barcelona cognitive battery), Free and Cued Selective Reminding Test, Boston Naming Test, Trail Making Test, Blessed Dementia Rating Scale and Alzheimer’s Disease Functional Assessment and Change Scale.

In the Brescia cohort^16^, SCD was defined as self-perceived cognitive decline with normal performance on neuropsychological testing, adjusted for age and education and without functional impairment (CDR = 0). MCI was diagnosed based on objective cognitive deficits in at least one cognitive domain on extensive neuropsychological evaluation, with CDR = 0.5. Dementia was defined as a cognitive complaint with objective deficits in at least one cognitive domain on neuropsychological tests (CDR > 1). The neuropsychological battery assessment used in the Brescia cohort included the following cognitive tests: MoCA for global cognition, memory tests (Short story, Free and Cued Selective Reminding test or RAVLT), semantic and phonetic fluency tasks, naming test (SAND battery for aphasia), Trail Making Test, Digit Span forward and backward, Clock drawing test, Rey Complex figure copy and recall, and CDR.

In the primary care cohort^9^, SCD was defined as experiencing cognitive symptoms to the level that led the patient to seek help in primary care, but not fulfilling the criteria for MCI or dementia. MCI was diagnosed in weekly consensus rounds, including a responsible dementia specialist and neuropsychologist, based on the presence of notable cognitive symptoms and abnormal cognitive test results using the RBANS (Repeatable Battery for the Assessment of Neuropsychological Status) battery (accounting for premorbid cognitive level) and the CDR. The MCI definition did not require that a strict threshold in a cognitive domain was met (although all performed <−1 s.d. in at least one cognitive domain in the RBANS battery), but was based on the overall clinical assessment. The classification followed the design of the MCI classification of the Mayo Clinic Study of Aging^29^ and was in line with the DSM-5 criteria for mild neurocognitive disorder^26^. Dementia was diagnosed according to the DSM-5 criteria for major neurocognitive disorder^26^.

### Plasma sampling and analysis

Plasma p-tau217 and Aβ42 were analyzed using the Lumipulse immunoassays (Fujirebio) at Gothenburg University, Sweden (the Malmö, Gothenburg and primary care cohorts), the Barcelonaβeta Brain Research Center, Spain (the Barcelona cohort) and the Department of Clinical Laboratory, ASST Spedali Civili Hospital, Italy (the Brescia cohort) in single batches. In the Malmö, Gothenburg, Brescia and primary care cohorts, p-tau217 and np-tau217 were also analyzed using MS-based assays at C2N Diagnostics, as previously described^14^. In addition to p-tau217, %p-tau217 (p-tau217:np-tau217 × 100) was also used. Additional details on plasma sampling and analyses are described in the Supplementary Information.

### Comorbidities and demographic variables

Diabetes was defined as being diagnosed with either diabetes type 1 or diabetes type 2 based on registered diagnoses in the medical records. CKD was defined as an estimated glomerular filtration rate <60 ml^−1^ min^−1^ 1.73 m^−2^. For age, participants were grouped as ≥80 years or <80 years. The <80-year group was further divided by a median split (73 years), yielding age categories <73 and 73–80.

### Establishing plasma cutoffs

The cutoffs were established in the Malmö cohort according to a previously published design^4^. The cutoff was set at 90% specificity for AD pathology with as high a sensitivity as possible (one-cutoff approach). In addition, a two-cutoff approach (using upper and lower cutoffs) was also established according to the Alzheimer’s Association Appropriate use recommendations for AD blood biomarkers^30^ and previous publications^1,9,31^, and similar to the FDA approval of the CSF Aβ42:40 Lumipulse assays^26^. The two cutoff values were set to achieve 95% sensitivity (with maximized specificity) and 95% specificity (with maximized sensitivity), respectively, in the Malmö cohort^4^. Results between these two cutoffs were termed ‘intermediate’. To quantify the expected variability of cutoffs in external samples (that is, robustness of cutoffs), we used a bootstrap procedure (*n* = 2,000 iterations) to estimate the 95% CI of all cutoffs. The means of all bootstrap samples were used as the specific cutoff points. The cutoff for plasma p-tau217 (Lumipulse) at 90% specificity was >0.27 (95% CI = 0.20–0.31) pg ml^−1^. The two-cutoff approach at 95% sensitivity and 95% specificity yielded a lower cutoff at <0.22 (95% CI = 0.17–0.25) pg ml^−1^ and an upper cutoff at >0.34 (95% CI = 0.30–0.41) pg ml^−1^ for Lumipulse p-tau217. In the same cohort, this approach was also used for establishing cutoffs for p-tau217:Aβ42 (Lumipulse) and the MS-based methods. The cutoff for p-tau217:Aβ42 at 90% specificity was >0.008 (95% CI = 0.007–0.009). For the two-cutoff approach, the lower 95% sensitivity cutoff was <0.007 (95% CI = 0.006–0.008) and the upper 95% specificity cutoff was >0.009 (95% CI = 0.008–0.011). For the MS-based measures, the mean cutoff for p-tau217 at 90% specificity was >2.27 (95% CI = 1.83–2.54) pg ml^−1^. For the two-cutoff approach, the lower cutoff at 95% sensitivity was <1.59 (95% CI = 0.74–1.85) pg ml^−1^ and the upper cutoff at 95% specificity was >2.92 (95% CI = 2.37–3.59) pg ml^−1^. For %p-tau217, this approach yielded >4.27 (95% CI = 3.44–4.99) pg ml^−1^ at 90% specificity, <3.55 (95% CI = 3.06-4.37) pg ml^−1^ at 95% sensitivity and >5.08 (95% CI = 4.56-5.68) pg ml^−1^ at 95% specificity. In a sensitivity analysis, the single cutoff was established in the Malmö cohort at the highest Youden index for AD pathology.

### Outcomes

The primary outcome was the presence of AD pathology, which was determined by the CSF Aβ42:p-tau181 ratio analyzed using the FDA-approved Lumipulse assays^26^. A cutoff of <11.94 for positivity was derived by unbiased Gaussian mixture modeling using all participants with CSF data (*n* = 1,679)^32^. For participants who could not undergo lumbar puncture in the BioFINDER-Primary Care study (*n* = 87), AD pathology was determined based on a positive visual read of [^18^F]flutemetamol PET. A sensitivity analysis was conducted, excluding individuals whose AD pathology was defined by amyloid PET. As a secondary outcome, the CSF Aβ42:40 ratio (Lumipulse) was used, with the FDA-approved cutoff of ≤0.072 indicating positivity^26^. Details of the CSF and PET analyses are described in the Supplementary Information.

### Statistical analysis

There were no missing Lumipulse p-tau217 values or outcome data (AD pathology) for the included participants. As none of the statistical analyses depended on the assumption of a normal data distribution, raw biomarker levels were used throughout all analyses and are presented in the figures. Binary variables were compared using χ^2^ tests, and continuous variables using the Mann–Whitney *U*-test. ROC curves were used to calculate the AUCs. AUCs were compared using the DeLong test. Significant differences across accuracies, PPVs and NPVs were examined using a bootstrap hypothesis test (*n* = 2,000 bootstrap samples). The 95% CIs were calculated using bootstrapping (*n* = 2,000 resamples with replacement) and *P* values for significant differences in test metrics (for example, accuracy) were calculated as the proportion of bootstrap resamples (*n* = 2,000), where the absolute null-distributed statistic was greater than or equal to the observed difference. In the two-cutoff approach, participants with an intermediate test result were not considered when calculating the accuracy, PPVs and NPVs, but have instead been specified in the figures. A two-sided *P* < 0.05 was considered to indicate a statistical significance. R programming language (https://www.R-project.org, v.4.4.2) was used for all statistical analyses with the following packages: dplyr (v.1.1.4), pROC (v.1.18.5), boot (v.1.331), cutpointr (v.1.1.2), readxl (v.1.4.3), tidyverse (v.2.0.0), ggplot2 (v.3.5.1) and ggpubr (v.0.6.0).

### Reporting summary

Further information on research design is available in the Nature Portfolio Reporting Summary linked to this article.

## Online content

Any methods, additional references, Nature Portfolio reporting summaries, source data, extended data, supplementary information, acknowledgements, peer review information; details of author contributions and competing interests; and statements of data and code availability are available at 10.1038/s41591-025-03622-w.

## Supplementary information

Supplementary Information Supplementary Methods, Tables 1–5 and Figs. 1–4.

Reporting Summary

## Data Availability

Anonymized data will be shared by request from a qualified academic investigator for the sole purpose of replicating procedures and results presented in the article, as long as data transfer is in agreement with EU legislation on the general data protection regulation and decisions by the ethical review board of each site, which should be regulated in a material transfer agreement.

## Code availability

The custom R codes for the analyses are available at GitHub (https://github.com/noellewarmenhoven/Lumipulse-Nat-Med).
