# Supplementary material for: Plasma phospho-tau217 for Alzheimer’s disease diagnosis in primary and secondary care using a fully automated platform
Source: Nat Med. 2025 Apr 9;31(6):2036–43. doi: 10.1038/s41591-025-03622-w (PMC12176611; doi:10.1038/s41591-025-03622-w)
Supplement: Supplementary file 1 — Supplementary Methods, Tables 1–5 and Figs. 1–4. [file 41591_2025_3622_MOESM1_ESM.pdf]

nature medicine

Supplementary information

https://doi.org/10.1038/s41591-025-03622-w

Plasma phospho-tau217 for Alzheimer's disease diagnosis in primary and secondary care using a fully automated platform

In the format provided by the authors and unedited

Online Supplement

Plasma phospho-tau217 for Alzheimer's disease diagnosis in primary and secondary care using a fully automated platform

Palmqvist, Warmenhoven, Anastasi et al.

Supplementary Methods 3
Lumipulse analysis of plasma p-tau217 4
Lumipulse analysis of plasma Aβ42 5
Mass spectrometry-based analysis of plasma p-tau217 and non p-tau217 5
CSF procedures 6
[18F]flutemetamol PET (amyloid PET) 6
Supplementary Tables 8
Supplementary Table 1. Plasma p-tau217 comparisons between primary and secondary care 8
Supplementary Table 2. Accuracy of plasma p-tau217 for identifying AD pathology stratified by age and cohort. 9
Supplementary Table 3. Additional characteristics of cohorts used in the comparison of Lumipulse plasma p-tau217 and p-tau217/Aβ42 10
Supplementary Table 4. Additional characteristics of cohorts used in the comparison of plasma Lumipulse and mass spectrometry-based assays. 11
Supplementary Table 5. Calculation of the costs savings for 1000 individuals by implementing a two cutoff-approach for plasma p-tau217 (Lumipulse) 12
Supplementary Figures 14
Supplementary Fig. 1. Shiny App for evaluating the cost-effectiveness of plasma p-tau217. A screenshot of the Shiny app is shown. 14
Supplementary Fig. 2. Performance of plasma p-tau217 (Lumipulse) for discriminating AD pathology positive versus AD pathology negative participants using CSF 15
Supplementary Fig. 3. Performance of plasma p-tau217 (Lumipulse) in primary care, excluding participants that underwent amyloid PET instead of CSF analysis. 16
Supplementary Fig. 4. Comparison of plasma p-tau217 (Lumipulse) cutoffs: 90% specificity vs. highest Youden Index for discriminating AD pathology. The accuracy (A), PPV (B), and NPV (C) are shown across all cohorts (n = 1767). 17

Supplementary Methods

Plasma collection

In the Malmö secondary care cohort and the primary care, blood was collected in EDTA tubes (BD Vacutainer; K2EDTA #367525) and centrifuged within 1 hour. The plasma was pipetted to 1 mL LoBind tubes (Sarstedt, #72.703.600) and frozen at -80°C before shipment to the Clinical Chemistry laboratory, Mölndal, Sweden for Lumipulse analyses and to C2N Diagnostics, St Louis, USA for mass spectrometry analyses. Samples were shipped on dry ice.

In the H70 Clinical Studies, blood was collected in EDTA tubes (BD Vacutainer 10 ml; K2EDTA #367525) and centrifuged within 1 hour. The plasma was pipetted to 1 mL LoBind tubes (Sarstedt, #72.703.600) and frozen at -80°C before shipment on dry ice to the Clinical Chemistry laboratory, Mölndal (Sweden) for Lumipulse analyses.

In the Barcelona cohort, blood samples were collected the same day of the lumbar puncture. Whole blood was drawn with a 20g or 21g needle gauge into a 10 ml EDTA tubes (BD Vacutainer 10 ml; K2EDTA; #367525). Tubes were gently inverted 5–10 times and centrifuged at 2000g for 10 minutes at (4ºC) within 1 hour. The supernatant was aliquoted in volumes of 1.8 ml into sterile polypropylene tubes (1.8 ml cryotube Thermo scientificTM NuncTM; Thermo Fisher Scientific, Waltham, MA, USA; #377267), and immediately frozen at −80°C. For this study, samples were shipped in dry ice to the Barcelonaβeta Brain Research Center (BBRC) Laboratory. Upon arrival, the samples were aliquoted into smaller volumes in polypropylene tubes and then frozen at −80ºC until measurements were performed

In the Brescia cohort, blood samples were collected in fasting condition using 9 mL EDTA tubes (K2EDTA; S-Monovette® EDTA Gel K2E; #04.1932.001). The tubes were gently inverted 5–10 times to mix the blood and then centrifuged at 2500×g for 10 minutes at room temperature. Next, 0.5mL plasma aliquots were pipetted into polypropylene cryotubes

(CLEARLine CryoGen Tubes, CL1ARBIPSTS) and stored at -80°C. For plasma analyses, samples were brought to room temperature (21–23 °C) and were centrifuged at 2000g for 5 minutes in the Central Laboratory of ASST Spedali Civili Hospital.

Lumipulse analysis of plasma p-tau217

The Lumipulse G pTau 217 Plasma assay, on a LUMIPULSE G instrument, is a specific 2-step set-up where the analyte is first captured in presence of an assay specific solution on RD85 coated particles, and after washing is detected with ALP labelled HT7/BT2 conjugate. The assay uses a synthetic peptide containing the three p-tau epitopes as the calibrator.

Plasma analyses for p-tau217 were performed blinded to all clinical and biomarker data and conducted in single batches cohort-wise. In the Malmö cohort, measurements were performed using the Lumipulse G600II, with samples run in singlicates over five days: March 14-15 and March 19-21, 2024. For the Gothenburg cohort, p-tau217 was measured using the Lumipulse G600II, with samples analyzed in singlicates over three days: March 21-23, 2024. In the Barcelona cohort, plasma p-tau217 measurements were conducted using the Lumipulse G p-tau217 plasma IRC RUO on a Lumipulse G1200 instrument, with samples run in singlicates on two days: February 7 and March 18, 2024. In the Brescia cohort, measurements were performed on the Lumipulse G600II, with samples analyzed in singlicates over seven days: March 4-6, 8, 15, 18, and 25, 2024. For the primary care cohort in Sweden, p-tau217 measurements were performed using the Lumipulse G1200, with samples analyzed in singlicates over three days: January 30, February 2, and February 9, 2024. All measurements were above the assay's lower limit of detection, except for two measurements in the Barcelona cohort.

Lumipulse analysis of plasma Aβ42

Plasma analyses for Aβ42 were measured with the Lumipulse G β-Amyloid 1-42 plasma assay, and were performed blinded to all clinical and biomarker data and conducted in single batches cohort-wise. In the Malmö cohort, measurements were performed using Lumipulse G600II on February 5 and February 7, 2024. For the Gothenburg cohort, measurements were performed using Lumipulse G600II on November 12 and 13, 2024. For the Barcelona cohort, measurements were performed using Lumipulse G1200 on June 6 and 15, 2023. For the Brescia cohort, measurements were performed using Lumipulse G600II from December 2022 to July 2023. For the primary care cohort in Sweden, measurements were performed using Lumipulse G1200 from December 2023 to March 2024.

Mass spectrometry-based analysis of plasma p-tau217 and non p-tau217

The plasma analyses were performed blinded to all clinical or biomarker data. In the Brescia and Gothenburg cohorts, the analysis was performed in single plasma batches. In the Malmö and primary care cohorts it was performed continuously during the study period in 31 batches. The measurements of p-tau217 and non p-tau217 in plasma isolated from blood samples has been described elsewhere.[1] Briefly, plasma samples were spiked with internal standards, and the analyte of interest was isolated from the sample prior to injection into the mass spectrometer. By combining the ratio of analyte to internal standard with similar ratios measured in standard curve samples, it is possible to calculate the concentration of the analyte of interest in the sample. The %p-tau217 was calculated as: p-tau217/np-tau217 * 100. For samples where the p-tau217 concentration fell below the limit of quantitation for the assay, but the mass spectrometer still detected both the internal p-tau217 standard and found a peak for p-tau217, the %p-tau217 was calculated using p-tau217 concentration imputed as ½ the limit of quantitation. All samples for this study were analyzed using the version 1 of the p-

tau217 assay developed at C2N Diagnostics (St. Louis, MO, USA). P-tau217 and %p-tau217 values were converted to p-tau217 version 2 and %p-tau217 version 2. In total, 177 samples fell below the limit of detection in secondary care, and 56 in primary care.

CSF procedures

In the Malmö and primary care cohorts, CSF was collected in 5 mL LoBind tubes (Eppendorf #0030108302) and aliquoted to 1.5 mL LoBind tubes (Sarstedt #72.703.600) and frozen at −80°C. In the Gothenburg cohort, CSF was collected in 10 mL polypropylene sterile tubes (Sarstedt # 62.9924.284) and aliquoted to 1.5 mL LoBind tubes (Sarstedt #72.703.600) and frozen at −80°C. In the Barcelona cohort, CSF was collected into a 10 ml sterile polypropylene sterile tube (Sarstedt #62.610.201) Tubes are gently inverted 5–10 times and centrifuged at 2000g for 10 minutes at room temperature and aliquoted in volumes of 0.5 ml into sterile polypropylene tubes (Sarstedt, Nümbrecht, Germany; #72.730.006) at Hospital del Mar's laboratory and immediately shipped with dry ice to Laboratori de Referència de Catalunya, where its frozen at −80°C until measurements are performed. In the Brescia cohort, CSF was collected either in 15 mL (Sarstedt #62.554.502) or in 5 mL (Sarstedt #63.504.027) polypropylene sterile tubes, gently mixed to avoid gradient effects, and sent directly (<30 minutes) to the hospital laboratory for routine assessments where the specimens were aliquoted to 2 mL tubes (Sarstedt #72.694.600) and frozen at -80°C.

CSF AD core biomarkers (Aβ42, Aβ40, and p-tau181) from all cohorts were measured using the Lumipulse® G assays (Fujirebio).

[18F]flutemetamol PET (amyloid PET)

In the primary care cohort, participants who were either unwilling or had contraindications for lumbar puncture underwent FDA-approved [^{18}F]flutemetamol PET

scans to assess the presence of Aβ pathology (n=87).[2] [^{18}F]flutemetamol scans were acquired in LIST mode using GE Discovery MI PET/CT cameras 90-110 minutes after injection of ~185 MBq [^{18}F]flutemetamol. Images were attenuation corrected using a low dose CT attenuation scan and were reconstructed using the GE-developed Q.Clear algorithm (BSREM - Block sequential regularized expectation maximization, with beta = 100). A 25.6-cm field of view (256 × 256 matrix) was used. Images were visually read according to guidelines, except that striatal retention was not evaluated.[3] In brief, images were assessed in a rainbow color scale where the retention in the pons/middle cerebellar peduncles was set to the yellow/red color shift. Cortical retention in the lateral temporal cortex, frontal cortex, precuneus and parietal lobes were assessed. Positivity in any one of these regions was considered enough for an overall positive scan. The final record was a dichotomous positive/negative read.

Supplementary Tables

Supplementary Table 1. Plasma p-tau217 comparisons between primary and secondary care.

**1 cutoff**	**Accuracy (95% CI)**	**PPV (95% CI)**	**NPV (95% CI)**	**AUC (95% CI)**
**Secondary care**	89 (88-91)*	92 (90-94)*	85 (82-88)	0.95 (0.94-0.96)*
**Primary care**	85 (82-88)	82 (77-87)	88 (84-91)	0.93 (0.90-0.95)
**2 cutoffs**	**Accuracy (95% CI)**	**PPV (95% CI)**	**NPV (95% CI)**	**%Intermediate (95% CI)**
**Secondary care**	94 (92-95)	95 (93-97)*	91 (89-94)	14 (13-17)
**Primary care**	92 (89-94)	90 (86-94)	92 (89-96)	16 (13-19)

Comparison between pooled secondary care (n = 1219) and primary care (n = 548) for discriminating AD pathology positive participants from AD pathology negative participants. The four secondary care cohorts were pooled together. Accuracy was defined as the percent correctly classified individuals. AD pathology was defined as CSF Aβ42/p-tau181 <11.94 or positive visual read on amyloid PET if lumbar puncture was not performed. Metrics were compared using bootstrapping. Abbreviations: accuracy, percentage correctly classified individuals; AUC, area under the curve; NPV: negative predictive value; PPV: positive predictive value.
* significantly larger than primary care ($p < 0.05$). *P* values in chronological order: 0.015; <0.001; 0.029; 0.029.

Supplementary Table 2. Accuracy of plasma p-tau217 for identifying AD pathology stratified by age and cohort.

Cohort	Accuracy, % (95% CI) Age < 73	Accuracy, % (95% CI) Age 73-80	Accuracy, % (95% CI) Age ≥ 80
**Malmö (n=337)**	95[c] (91-98)	88 (82-93)	80 (70-90)
**Gothenburg (n=165)**	93 (87-99)	91 (85-97)	60 (0-100)
**Barcelona (n=487)**	91 (87-95)	88 (84-92)	87 (67-100)
**Brescia (n=230)**	88 (81-94)	89 (83-94)	91 (77-100)
**Pooled secondary (n=1219)**	91[c] (88-93)	89 (87-92)	82 (74-89)
**Primary care (n=548)**	88 (83-92)	84 (79-89)	84 (78-89)
**Pooled secondary, no CKD (n=1027)**	91 (88-93)[c]	89 (87-92)	79 (68-89)
**Primary care, no CKD (n=382)**	88 (83-93)	82 (75-87)	80 (71-89)

Accuracy of plasma p-tau217 (Lumipulse) for discriminating AD pathology positive participants from AD pathology negative participants across the three age categories, separated by cohort. Results are shown using a single cutoff. Accuracy was defined as the percent correctly classified individuals. AD pathology was defined as CSF Aβ42/p-tau181 <11.94 or positive visual read on amyloid PET if lumbar puncture was not performed. Accuracies were compared using bootstrapping. *P* values in chronological order: 0.004; 0.029; 0.046.
[c] significantly higher accuracy than the >80 years group ($p < 0.05$).

Supplementary Table 3. Additional characteristics of cohorts used in the comparison of Lumipulse plasma p-tau217 and p-tau217/Aβ42.

Variable	Secondary care (pooled) n=911	Primary care (Sweden) n=502
**Secondary care cohort (Malmö, Gothenburg, Barcelona, Brescia), n**	337/164/356/54	n/a
**Age, y**	71 (8.3)	76 (6.9)
**Sex, n female**	478 (52.5%)	257 (51.2%)
**Education, years**	11 (4.4)	11 (3.2)
**Diabetes, n**	165 (18.1%)	108 (21.5%)
**Chronic kidney disease, n**	102 (11.2%)	120 (23.9%)
**MMSE**	23 (5.3)	26 (3.4)
**Cognitive stage, n (%)**		
**SCD**	94 (10.3%)	140 (27.9%)
**MCI**	445 (48.8%)	227 (45.2%)
**Dementia**	372 (40.8%)	135 (26.9%)
***APOE* ε4 carriers, n (%)**	410 (45.0%)	220 (43.8%)
**Plasma p-tau217 pg/mL**	0.47 (0.44)	0.38 (0.37)
**Plasma p-tau217/Aβ42 ratio**	0.025 (0.05)	0.014 (0.01)
**CSF Aβ42/p-tau181 ratio**	16 (13.9)	19 (12.9)[1]
**AD positive, n (%)[2]**	528 (58.0%)	220 (43.8%)

[1] CSF missing for n = 83 in the primary care cohort.

[2] CSF Aβ42/p-tau181 <11.94 or positive amyloid PET visual read for those who did not undergo lumbar puncture (only in the primary care cohort). Comorbidity and demographical data were missing for <7% in the total sample. There were no missing biomarker data. Abbreviations: AD, Alzheimer's disease; MCI, mild cognitive impairment; MMSE, Mini-Mental State Examination; SCD, subjective cognitive decline.

Supplementary Table 4. Additional characteristics of cohorts used in the comparison of plasma Lumipulse and mass spectrometry-based assays.

**Variable**	**Secondary care (pooled) n=619**	**Primary care (Sweden) n=513**
**Secondary care cohort (Malmö, Gothenburg, Brescia), n**	337/164/118	n/a
**Age, y**	71 (9.0)	76 (6.9)
**Sex, n female**	304 (49.1%)	257 (50.1%)
**Education, years**	12 (3.9)	11 (3.2)
**Diabetes, n**	78 (12.6%)	111 (21.6%)
**Chronic kidney disease, n**	121 (19.5%)	127 (24.8%)
**MMSE**	24 (4.8)	26 (3.4)
**Cognitive stage, n (%)**		
**SCD**	69 (11.1%)	140 (27.3%)
**MCI**	368 (59.5%)	234 (45.6%)
**Dementia**	182 (29.4%)	139 (27.1%)
***APOE* ε4 carriers, n (%)**	253 (40.9%)	219 (42.7%)
**Plasma p-tau217 pg/mL (Lumipulse)**	0.42 (0.41)	0.38 (0.36)
**Plasma p-tau217, pg/mL (mass spectrometry)**	3.3 (3.5)	3.8 (4.0)
**Plasma %p-tau217 (mass spectrometry)**	5.8 (5.4)	5.9 (5.6)
**CSF Aβ42/40 ratio**	0.070 (0.046)[1]	0.069 (0.026)[2]
**CSF p-tau181, pg/mL**	67 .1 (45.5)	64.3(41.0)
**CSF Aβ42/p-tau181 ratio**	18.3 (17.3)	18.7 (13.1)
**AD positive, n (%)[3]**	330 (53.3%)	226 (44.1%)

[1] CSF data is missing for n = 9.

[2] CSF data is missing for n = 80 in the primary care cohort only.

[3] CSF Aβ42/p-tau181 <11.94 or positive amyloid PET visual read for those who did not undergo lumbar puncture (only in the primary care cohort). Comorbidity and demographical data were missing for <5% in the total sample. There were no missing biomarker data. Mass spectrometry-based assays were not available in the Barcelona cohort. Abbreviations: AD, Alzheimer's disease; MCI, mild cognitive impairment; MMSE, Mini-Mental State Examination; SCD, subjective cognitive decline.

Supplementary Table 5. Calculation of the costs savings for 1000 individuals by implementing a two cutoff-approach for plasma p-tau217 (Lumipulse)

		**Using CSF**	**Using PET**
		1000 USD	6000 USD
**Plasma p-tau217 (Lumipulse)**		250 USD	
**Standard workflow**	**Blood test**<br>**CSF or PET**	-<br>1,000,000	-<br>6,000,000
	**Total**	*1,000,000*	*6,000,000*
**Two-step approach** (plasma p-tau217, followed by CSF or PET if intermediate plasma p-tau217 result)	**Blood test** (p-tau217)	250,000	250,000
	**Follow-up with CSF or PET** (intermediate p-tau217 values)	147,000	882,000
	**Total**	*397,000*	*1,132,000*
**Cost saving**		60.3%	81.1%

The table compares the costs of the standard approach, where all participants undergo either CSF or amyloid PET testing, with the two-step workflow. In the two-step workflow, all participants first undergo a plasma p-tau217 test, and only those with intermediate results proceed to CSF or amyloid PET testing. The approximate costs per patient were estimated as follows: $1000 for CSF testing, $6000 for amyloid PET, and $250 for plasma p-tau217 (Lumipulse) testing. The bottom row of the table highlights the potential cost savings of the two-step approach under various cost scenarios. Adopting the two-step workflow can achieve savings of up to 60.4% compared to CSF-only workflows and up to 81.2% compared to amyloid PET-only workflows. Since test costs vary by country and center, an online calculator is available to estimate cost savings based on local costs: https://bbrc-lab.shinyapps.io/Cost-effectiveness_analysis_plasma_p-Tau217. Supplementary Fig. 1 shows an example.

Supplementary Figures

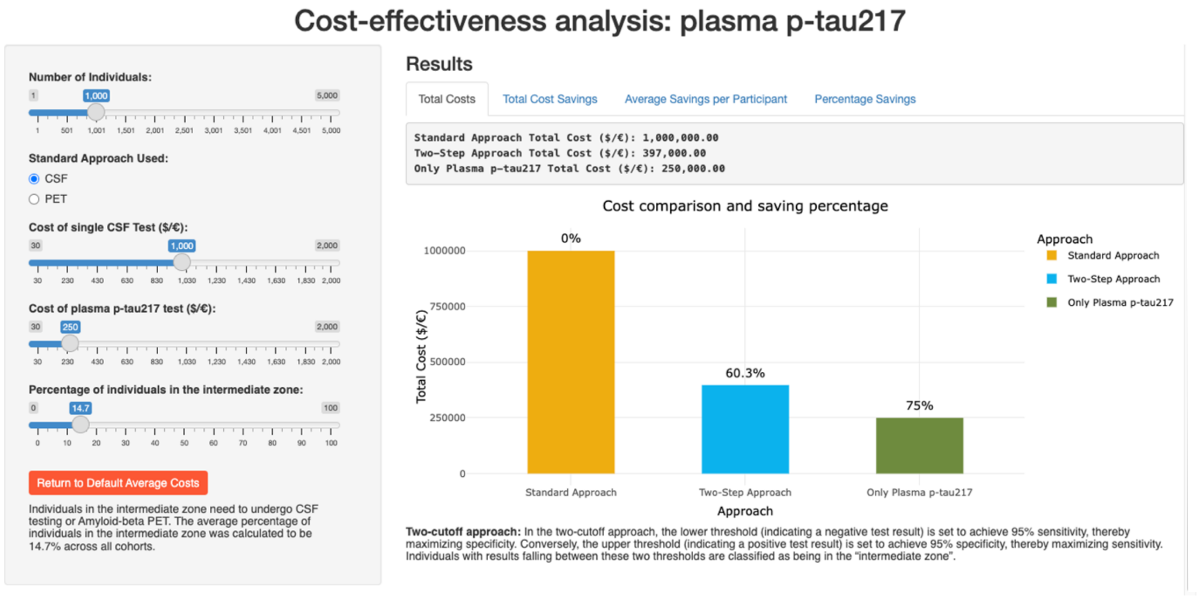

Supplementary Fig. 1. Shiny App for evaluating the cost-effectiveness of plasma p-tau217. A screenshot of the Shiny app is shown. This interactive application was developed to assess the cost-effectiveness of using plasma p-tau217 (Lumipulse) compared to standard approaches, namely CSF analysis or amyloid PET scans. The app allows users to input site-specific costs and calculate potential cost savings using either the two-step or the one-step approach both based on plasma p-tau217 with respect to the standard approaches. Additionally, the app provides options to visualize and download analysis results as PNG plots for customized reporting. The app can be accessed at https://bbrc-lab.shinyapps.io/Cost-effectiveness_analysis_plasma_p-Tau217.

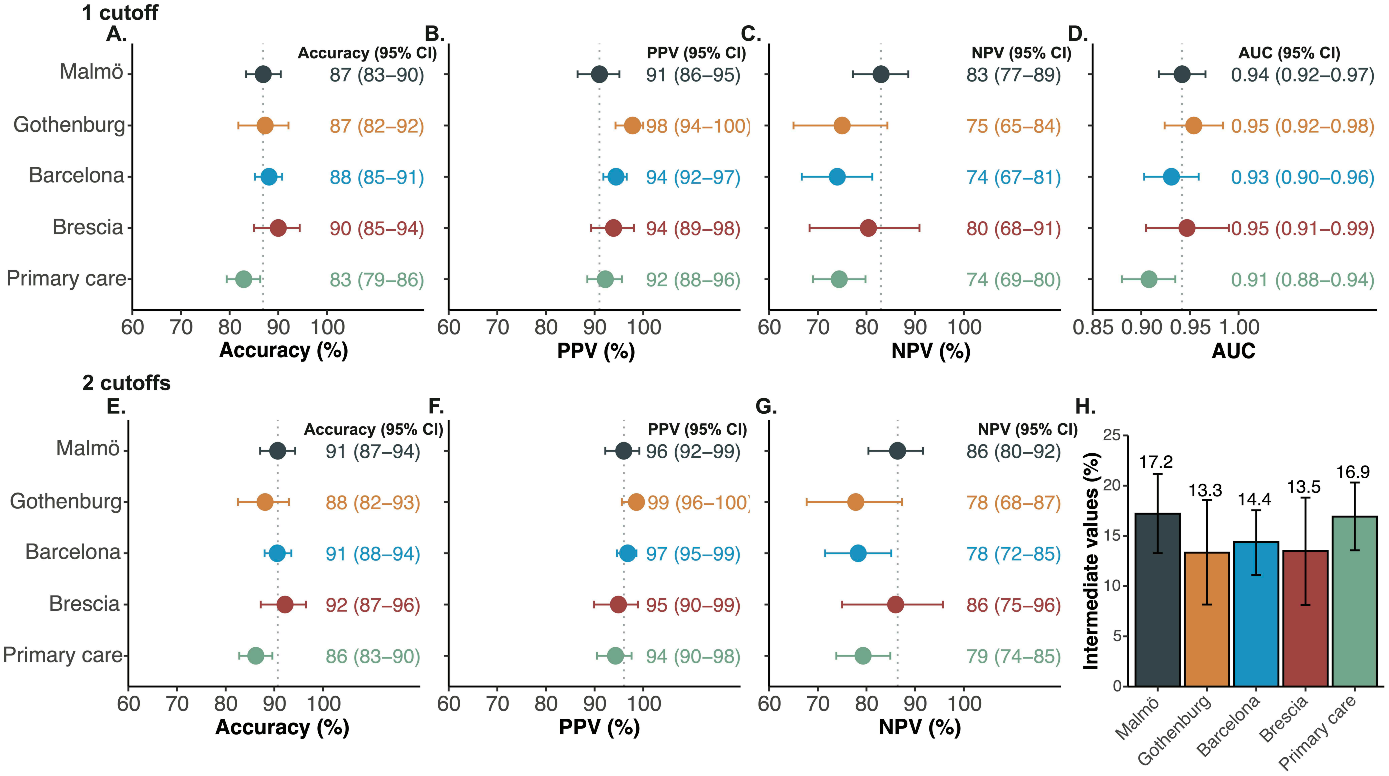

Supplementary Fig. 2. Performance of plasma p-tau217 (Lumipulse) for discriminating AD pathology positive versus AD pathology negative participants using CSF Aβ42/40 as a reference standard. Cutoffs were established in the Malmö secondary care cohort using the FDA-approved Lumipulse CSF Aβ42/40 assay. A cutoff of ≤0.072 was considered positive, as previously described. The single cutoff was set at >0.24 pg/mL (A-C) and the two cutoffs at <0.16 and >0.30 pg/mL (E-G). Note that the AUC values (D) are independent of cutoffs. Participants who fall between the two cutoffs were classified in the intermediate group (H). Vertical dashed lines mark the performance in the Malmö cohort where the cutoffs were established. The dots or bars represent the actual percentage, and the error bars the 95% CI derived from the bootstrap distribution. The AD pathology prevalence was *n* =180+/157- in Malmö, 106+/59- in Gothenburg, 357+/130- in Barcelona, 116+/44- in Brescia, and 264+/197- in primary care (Sweden). CSF Aβ42/40 data was missing for *n* = 70 in the Brescia cohort, and *n* = 87 in the primary care cohort. Abbreviations: Accuracy, percent correctly classified participants; CI; confidence interval; NPV, negative predictive value; PPV, positive predictive value.

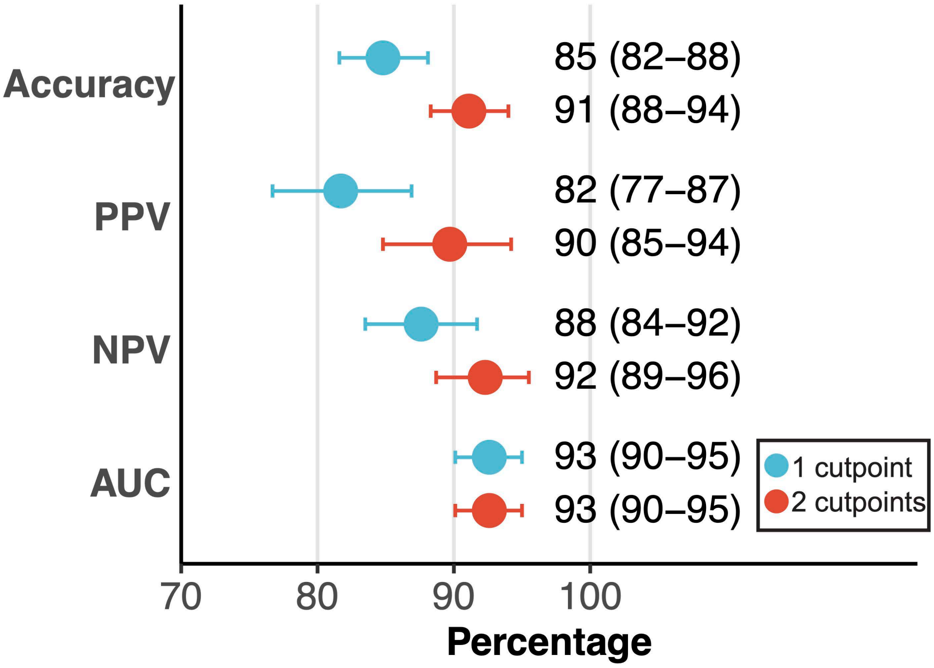

Supplementary Fig. 3. Performance of plasma p-tau217 (Lumipulse) in primary care, excluding participants that underwent amyloid PET instead of CSF analysis. The figure presents the accuracy, predictive values and AUCs of plasma p-tau217 in the primary care cohort, excluding participants without CSF status available (n = 87), yielding a sample size of n = 461. Results are shown using a single cutoff (blue) or using two cutoffs (red). The dots represent the actual percentage and the error bars the 95% CI derived from the bootstrap distribution**.** Abbreviations: Accuracy; percent correctly classified participants; CI, confidence intervals; NPV, negative predictive value; PPV, positive predictive value.

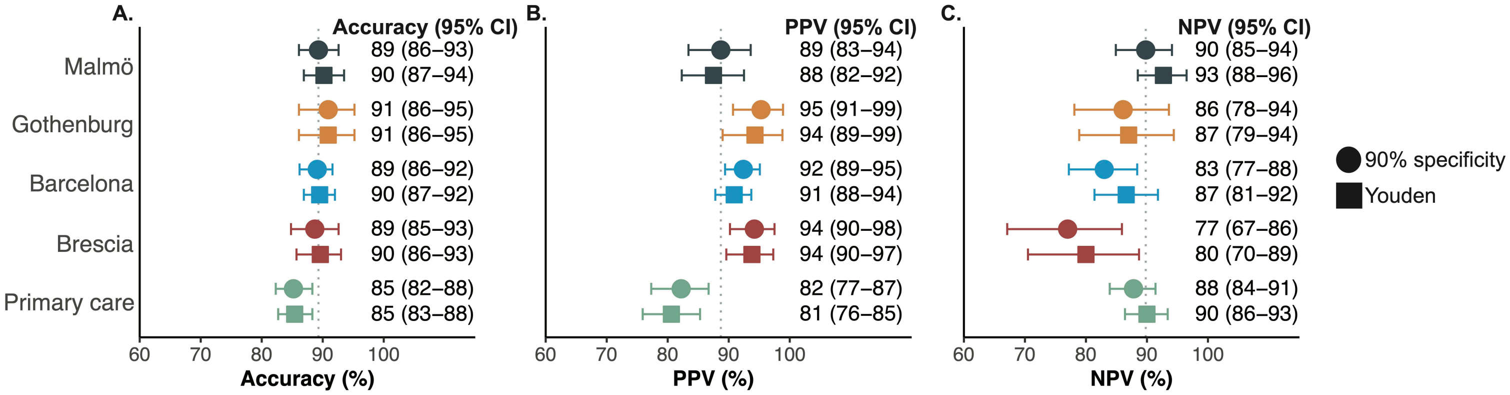

Supplementary Fig. 4. Comparison of plasma p-tau217 (Lumipulse) cutoffs: 90% specificity vs. highest Youden Index for discriminating AD pathology. The accuracy (A), PPV (B), and NPV (C) are shown across all cohorts (*n* = 1767). The cutoff at 90% specificity was set at >0.27 pg/mL and the cutoff at the highest Youden index >0.25 pg/mL using AD pathology as outcome. Note that the AUC values are independent of cutoffs and are therefore not included here. Vertical dashed lines mark the performance for the 90% specificity cutoff in the Malmö cohort where the cutoffs were established. The dots represent the actual percentage, and the error bars the 95% CI derived from the bootstrap distribution. Abbreviations: Accuracy, percent correctly classified participants; CI; confidence interval; NPV, negative predictive value; PPV, positive predictive value.

ONLINE SUPPLEMENT REFERENCES

1 Rissman, R. A. *et al.* Plasma Abeta42/Abeta40 and phospho-tau217 concentration ratios increase the accuracy of amyloid PET classification in preclinical Alzheimer's disease. *Alzheimers Dement* (2023). https://doi.org:10.1002/alz.13542

2 Palmqvist, S. *et al.* Discriminative Accuracy of Plasma Phospho-tau217 for Alzheimer Disease vs Other Neurodegenerative Disorders. *JAMA* **324**, 772-781 (2020). https://doi.org:10.1001/jama.2020.12134

3 Buckley, C. J. *et al.* Validation of an electronic image reader training programme for interpretation of [18F]flutemetamol beta-amyloid PET brain images. *Nucl Med Commun* **38**, 234-241 (2017). https://doi.org:10.1097/MNM.0000000000000633
