# Supplementary material for: Plasma phospho-tau217 for Alzheimer’s disease diagnosis in primary and secondary care using a fully automated platform
Source: Nat Med. 2025 Apr 9;31(6):2036–43. doi: 10.1038/s41591-025-03622-w (PMC12176611; doi:10.1038/s41591-025-03622-w)
Supplement: Supplementary file 2 — Reporting Summary [file 41591_2025_3622_MOESM2_ESM.pdf]

nature portfolio

Corresponding author(s): Sebastian Palmqvist
Oskar Hansson

Last updated by author(s): Jan 23, 2025

Reporting Summary

Nature Portfolio wishes to improve the reproducibility of the work that we publish. This form provides structure for consistency and transparency in reporting. For further information on Nature Portfolio policies, see our Editorial Policies and the Editorial Policy Checklist.

Statistics

For all statistical analyses, confirm that the following items are present in the figure legend, table legend, main text, or Methods section.

n/a	Confirmed	
☐	☒	The exact sample size (*n*) for each experimental group/condition, given as a discrete number and unit of measurement
☐	☒	A statement on whether measurements were taken from distinct samples or whether the same sample was measured repeatedly
☐	☒	The statistical test(s) used AND whether they are one- or two-sided<br>*Only common tests should be described solely by name; describe more complex techniques in the Methods section.*
☐	☒	A description of all covariates tested
☐	☒	A description of any assumptions or corrections, such as tests of normality and adjustment for multiple comparisons
☐	☒	A full description of the statistical parameters including central tendency (e.g. means) or other basic estimates (e.g. regression coefficient) AND variation (e.g. standard deviation) or associated estimates of uncertainty (e.g. confidence intervals)
☐	☒	For null hypothesis testing, the test statistic (e.g. *F*, *t*, *r*) with confidence intervals, effect sizes, degrees of freedom and *P* value noted<br>*Give P values as exact values whenever suitable.*
☒	☐	For Bayesian analysis, information on the choice of priors and Markov chain Monte Carlo settings
☒	☐	For hierarchical and complex designs, identification of the appropriate level for tests and full reporting of outcomes
☒	☐	Estimates of effect sizes (e.g. Cohen's *d*, Pearson's *r*), indicating how they were calculated

Our web collection on statistics for biologists contains articles on many of the points above.

Software and code

Policy information about availability of computer code

Data collection	No software was used for data collection.
Data analysis	All analyses were done using R version 4.4.2 with packages: dplyr (version 1.1.4), pROC (version 1.18.5), boot (version 1.331), cutpointr (version 1.1.2), readxl (version 1.4.3), tidyverse (version 2.0.0), ggplot2 (version 3.5.1), ggpubr (version 0.6.0).<br>Codes can be found on GitHub: https://github.com/noellewarmenhoven/Lumipulse-Nat-Med.

For manuscripts utilizing custom algorithms or software that are central to the research but not yet described in published literature, software must be made available to editors and reviewers. We strongly encourage code deposition in a community repository (e.g. GitHub). See the Nature Portfolio guidelines for submitting code & software for further information.

Data

Policy information about availability of data

All manuscripts must include a data availability statement. This statement should provide the following information, where applicable:

- Accession codes, unique identifiers, or web links for publicly available datasets
- A description of any restrictions on data availability
- For clinical datasets or third party data, please ensure that the statement adheres to our policy

Anonymized data will be shared by request from a qualified academic investigator for the sole purpose of replicating procedures and results presented in the article

and as long as data transfer is in agreement with EU legislation on the general data protection regulation and decisions by the Ethical Review Board of each site, which should be regulated in a material transfer agreement.

Research involving human participants, their data, or biological material

Policy information about studies with human participants or human data. See also policy information about sex, gender (identity/presentation), and sexual orientation and race, ethnicity and racism.

Reporting on sex and gender	The study includes both men and women. Sex was assigned (not self reported). 930 (53%) were female. Results are reported separately for men and women
Reporting on race, ethnicity, or other socially relevant groupings	The study does not include any references to race, ethnicity, or other socially relevant groupings.
Population characteristics	Detailed information is given in Table 1 for each cohort separately and in the Methods. In short, we present results for analyses from five different cohorts with very similar demographics. Participants presenting memory complaints were included in the present study (n=1,767) with cross-sectional. Among those, 250 had subjective cognitive decline (SCD), 858 had MCI and 658 had dementia. The mean (SD) age was 70 (8.8) years and 69% (n=698) were women. 975 (55%) were AD pathology positive. The mean age was 72 and 53% were women.
Recruitment	All participants in all five studies were recruited as part of undergoing a memory investigation in clinical practice to ensure a representative, real-life study population.<br><br>Malmö cohort: the inclusion criteria are: 1) being under investigation for cognitive symptoms at the Memory Clinic of Skåne University Hospital, Sweden; 2) CSF and blood sampling is planned to be done as part of clinical practice even if the patient is not taking part of this study. The exclusion criteria are 1) not undergoing CSF or blood sampling as part of clinical practice and 2) not undergoing cognitive testing as part of clinical practice. Patients included in this study were consecutively recruited between Dec 2022 and Nov 2023.<br><br>Gothenburg cohort: the inclusion criteria are: to be under investigation for cognitive symptoms at the Memory Clinic of Sahlgrenska University Hospital, Sweden. There are no exclusion criteria. For this study, participants with CSF and blood sampling as part of the clinical investigation were included. Patients in this study were consecutively recruited between Mar 2020 and Jun 2023.<br><br>Barcelona cohort: the inclusion criteria are: (i) undergoing evaluation at the Cognitive and Behavioural Neurology Unit and inclusion in the DEGMAR register; (ii) signed informed consent; and (iii) having one of the following clinical diagnoses: SCD, MCI, AD dementia; behavioural variant frontotemporal dementia (bvFTD); progressive aphasia or primary progressive aphasia (logopenic, non-fluent and semantic variants, PA); Lewy body dementia (LBD); corticobasal syndrome (CBS); progressive supranuclear palsy syndrome (PSP-S) and vascular cognitive impairment and dementia (VCID). Individuals with other causes of dementia but unspecified clinical diagnoses were also included and categorized as “other”. The exclusion criteria are: (i) age ≥80 years; (ii) contraindication for lumbar puncture; or (iii) disagreement with study procedures. Patients included in this study were consecutively recruited between Apr 2017 and Nov 2023.<br><br>Brescia cohort: the inclusion criteria are: participants with MCI or mild dementia who underwent clinical routine CSF assessment at the outpatient Neurodegenerative clinic of the Brescia University Hospital, Italy. The following exclusion criteria were applied: 1) cortical or subcortical cerebrovascular infarcts in structural imaging; 2) other neurologic disorders or medical conditions potentially associated with cognitive deficits; 3) bipolar disorder, schizophrenia, history of drug or alcohol abuse or impulse control disorder; 4) recent traumatic events or acute fever/inflammation; and 5) refusal of collection of blood sampling for research purposes. Patients in this study were consecutively recruited between Mar 2020 and Nov 2023.<br><br>Primary care cohort: the inclusion criteria are: 1) patient seeks medical help in primary care because of cognitive symptoms experienced by the patient or informant, or the primary care physician (PCP) suspects a neurodegenerative disorder; 2) age ≥40 years; and 3) cognitive impairment characterized as SCD, MCI or mild dementia. The exclusion criteria are 1) already diagnosed dementia; 2) significant unstable systemic illness making it difficult to participate in the study; 3) current significant alcohol or substance misuse; 4) refusing investigation at the memory clinic; 5) cognitive impairment with acute onset due to stroke; and 6) the cognitive impairment can with high certainty, as assessed by the primary care physician, be explained by another condition or disease such as psychotic disorder, depression, alcohol abuse etc. Patients included in this study were consecutively recruited from 19 primary care units in the south of Sweden from Jan 2020 to Nov 2023.
Ethics oversight	The studies were approved by the Swedish Ethical Review Authority (the Malmö, Gothenburg, and Primary Care cohorts), the Independent Ethics Committee “Parc de Salut Mar” [illegible]Barcelona, Spain (the Barcelona cohort), and the Spedali Civili of Brescia local ethics committee, Italy (the Brescia cohort).

Note that full information on the approval of the study protocol must also be provided in the manuscript.

Field-specific reporting

Please select the one below that is the best fit for your research. If you are not sure, read the appropriate sections before making your selection.

☒ Life sciences ☐ Behavioural & social sciences ☐ Ecological, evolutionary & environmental sciences

Life sciences study design

All studies must disclose on these points even when the disclosure is negative.

Sample size	The study included a large sample size (n=1,767) participants with cross-sectional data across five different cohorts. In several analyses, the data has been pooled to increase statistical power.
Data exclusions	Analyses were performed on all eligible participants and not on a restricted sample with complete data for all cognitive and non-cognitive measures. The rationale for this was to not introduce a selection bias. When participants have been removed for better visualization, the data has been extensively described in the footnotes and included in the statistical analyses.
Replication	Five independent cohorts were used in the study in the main analyses. Data was pooled to achieve better statistical power in sub-analyses. Replication of the results, when applicable, was successful.
Randomization	In these 5 cohort studies (observational studies) no allocation into experimental groups were performed, therefore randomization is not relevant to this study. The potential confounding effects of covariates has been investigated as part of the main analysis.
Blinding	Diagnostic assessments and all test measures were performed blinded to the plasma biomarker results.

Reporting for specific materials, systems and methods

We require information from authors about some types of materials, experimental systems and methods used in many studies. Here, indicate whether each material, system or method listed is relevant to your study. If you are not sure if a list item applies to your research, read the appropriate section before selecting a response.

Materials & experimental systems

n/a	Involved in the study	
☐	☒	Antibodies
☒	☐	Eukaryotic cell lines
☒	☐	Palaeontology and archaeology
☒	☐	Animals and other organisms
☐	☒	Clinical data
☒	☐	Dual use research of concern
☒	☐	Plants

Methods

n/a	Involved in the study	
☒	☐	ChIP-seq
☒	☐	Flow cytometry
☒	☐	MRI-based neuroimaging

Antibodies

Antibodies used	Lumipulse Phosphorylated tau-217 (ver 1) and amyloid-beta 42 (ver 1).
Validation	Plasma p-tau217 and Aβ42 were analyzed using the Lumipulse immunoassays (Fujirebio, Belgium) at Gothenburg University, Sweden (the Malmö, Gothenburg, and Primary Care cohorts), at the Barcelonabeta Brain Research Center, Spain (the Barcelona cohort) and the Department of Clinical Laboratory, ASST Spedali Civili Hospital, Italy (the Brescia cohort) in single batches. In the Malmö, Gothenburg, Brescia, and primary care cohorts, p-tau217 and non-p-tau217 were also analyzed using mass spectrometry-based assays at C2N Diagnostics, USA, as described in Meyer et al. (Alzheimers Dement. 2024;20:3179-3192).

Clinical data

Policy information about clinical studies

All manuscripts should comply with the ICMJE guidelines for publication of clinical research and a completed CONSORT checklist must be included with all submissions.

Clinical trial registration	Malmö cohort NCT06122415. Primary care cohort: NCT06120361.
Study protocol	Malmö cohort: https://biofinder.se/memory-clinic/<br>Gothenburg cohort: https://www.gu.se/en/research/h70-clinical-studies<br>Barcelona cohort: PMID: 36370462 and PMID: 35961506<br>Brescia cohort: PMID 39679606<br>Primary care cohort: https://biofinder.se/primare-care/
Data collection	All participants in all five studies were recruited as part of undergoing a memory investigation in clinical practice to ensure a representative, real-life study population.<br><br>Malmö cohort: data collected at the Memory Clinic of Skåne University Hospital, Sweden between Dec 2022 and Nov 2023.

	Gothenburg cohort: data collected at the Memory Clinic of Sahlgrenska University Hospital, Sweden between Mar 2020 and Jun 2023.<br><br>Barcelona cohort: data collected at the Cognitive and Behavioural Neurology Unit at Hospital del Mar in Barcelona, Spain between Apr 2017 and Nov 2023.<br><br>Brescia cohort: data collected at the outpatient Neurodegenerative clinic of the Brescia University Hospital, Italy between Mar 2020 and Nov 2023.<br><br>Primary care cohort: data collected from 19 different primary care institutions in Skåne, Sweden, from Jan 2020 to Nov 2023.
Outcomes	The primary outcome was AD status defined as a positive amyloid CSF test result or amyloid-PET test result.

Plants

Seed stocks	-
Novel plant genotypes	-
Authentication	-
